# School water, sanitation, and hygiene (WaSH) intervention to improve malnutrition, dehydration, health literacy, and handwashing: a cluster-randomised controlled trial in Metro Manila, Philippines

**DOI:** 10.1186/s12889-022-14398-w

**Published:** 2022-11-07

**Authors:** Stephanie O. Sangalang, Allen Lemuel G. Lemence, Zheina J. Ottong, John Cedrick Valencia, Mikaela Olaguera, Rovin James F. Canja, Shyrill Mae F. Mariano, Nelissa O. Prado, Roezel Mari Z. Ocaña, Patricia Andrea A. Singson, Ma. Lourdes Cumagun, Janine Liao, Maria Vianca Jasmin C. Anglo, Christian Borgemeister, Thomas Kistemann

**Affiliations:** 1 Center for Development Research, University of Bonn, Genscherallee 3, 53113 Bonn, Germany; 2 Department of Industrial Engineering, University of the Philippines Los Baños, Los Baños, Philippines; 3 School of Earth Sciences and Environmental Engineering, Gwangju Institute of Science and Technology, Gwangju, South Korea; 4 National Institute of Physics, College of Science, University of the Philippines Diliman, Quezon City, Philippines; 5 Philippines Department of Education, Meralco Avenue, Pasig City, Philippines; 6 College of Mass Communication, University of the Philippines Diliman, Quezon City, Philippines; 7 Marine Science Institute, University of the Philippines Diliman, Quezon City, Philippines; 8 Department of Environment Systems, University of Tokyo, Kashiwa, Chiba Japan; 9 National Institute of Geological Sciences, University of the Philippines Diliman, Quezon City, Philippines; 10 School of Medicine, Far Eastern University - Nicanor Reyes Medical Foundation, Quezon City, Philippines; 11 School of Social Sciences, Ateneo de Manila University, Quezon City, Philippines; 12 Department of Science and Technology, Food and Nutrition Research Institute, Taguig, Philippines; 13 School of Diplomacy and Governance, De La Salle - College of Saint Benilde, Manila, Philippines; 14 Department of Psychology, University of the Philippines Diliman, Quezon City, Philippines; 15 Institute of Hygiene and Public Health, University of Bonn, Bonn, Germany

**Keywords:** Child malnutrition, Dehydration, Health literacy, Water, Sanitation, Hygiene

## Abstract

**Background:**

The impacts of multicomponent school water, sanitation, and hygiene (WaSH) interventions on children’s health are unclear. We conducted a cluster-randomized controlled trial to test the effects of a school WaSH intervention on children’s malnutrition, dehydration, health literacy (HL), and handwashing (HW) in Metro Manila, Philippines.

**Methods:**

The trial lasted from June 2017 to March 2018 and included children, in grades 5, 6, 7, and 10, from 15 schools. At baseline 756 children were enrolled. Seventy-eight children in two clusters were purposively assigned to the control group (CG); 13 clusters were randomly assigned to one of three intervention groups: low-intensity health education (LIHE; two schools, n = 116 children), medium-intensity health education (MIHE; seven schools, n = 356 children), and high-intensity health education (HIHE; four schools, n = 206 children). The intervention consisted of health education (HE), WaSH policy workshops, provision of hygiene supplies, and WaSH facilities repairs. Outcomes were: height-for-age and body mass index-for-age Z scores (HAZ, BAZ); stunting, undernutrition, overnutrition, dehydration prevalence; HL and HW scores. We used anthropometry to measure children’s physical growth, urine test strips to measure dehydration, questionnaires to measure HL, and observation to measure HW practice. The same measurements were used during baseline and endline. We used multilevel mixed-effects logistic and linear regression models to assess intervention effects.

**Results:**

None of the interventions reduced undernutrition prevalence or improved HAZ, BAZ, or overall HL scores. Low-intensity HE reduced stunting (adjusted odds ratio [aOR] 0.95; 95% CI 0.93 to 0.96), while low- (aOR 0.57; 95% CI 0.34 to 0.96) and high-intensity HE (aOR 0.63; 95% CI 0.42 to 0.93) reduced overnutrition. Medium- (adjusted incidence rate ratio [aIRR] 0.02; 95% CI 0.01 to 0.04) and high-intensity HE (aIRR 0.01; 95% CI 0.00 to 0.16) reduced severe dehydration. Medium- (aOR 3.18; 95% CI 1.34 to 7.55) and high-intensity HE (aOR 3.89; 95% CI 3.74 to 4.05) increased observed HW after using the toilet/urinal.

**Conclusion:**

Increasing the intensity of HE reduced prevalence of stunting, overnutrition, and severe dehydration and increased prevalence of observed HW. Data may be relevant for school WaSH interventions in the Global South. Interventions may have been more effective if adherence was higher, exposure to interventions longer, parents/caregivers were more involved, or household WaSH was addressed.

**Trial registration number:**

DRKS00021623.

**Supplementary information:**

The online version contains supplementary material available at 10.1186/s12889-022-14398-w.

## Background

About one in three people suffer from malnutrition globally [1]. The severity of malnutrition’s consequences ranges from mild and temporary (e.g., slight weight loss) to severe and long-lasting (e.g., muscle wasting, impaired cardiorespiratory function). Malnutrition’s social consequences (e.g., decreased education attainment, reduced income) are grave because of long-term impacts on communities, which may experience increasing intergenerational vulnerability to disease and deprivation [2]. Malnutrition is harmful to countries because it slows economic growth and perpetuates poverty [3]. Annually, malnutrition costs 5% of the global gross domestic product (GDP) (United States Dollar [USD] ~ 3.5 trillion) [4]. In low- and middle-income countries (LMICs), a “double burden of malnutrition” may be found, where multiple forms of malnutrition (e.g. undernutrition and obesity) co-exist in the same household, community, or population [1]. The double burden of malnutrition, with its associated non-communicable diseases (NCDs), e.g. cardiovascular disease, diabetes, may soon overwhelm already constrained health systems and cause serious economic costs that hinder development and poverty eradication in LMICs. Such setbacks could have global consequences.

Although dehydration’s consequences (e.g., dry mouth, headache) seem benign, it can become a medical emergency characterized by tachypnoea and tachycardia. In 2004, dehydration caused 518,000 hospitalizations in the United States of America (U.S.), resulting in USD 5.5 billion worth of hospital charges [5]. Very young and very old individuals have greater risks for dehydration due to impaired fluid regulation. However, school-age children should not be ignored because dehydration’s negative impacts on cognitive function and mood [6] could increase school absenteeism and dropout.

Health literacy (HL) is the ability to understand concepts and practice behaviours that promote one’s well-being and prevent illness [7]. Low HL is problematic because it is associated with unhealthy behaviors [8] and poor health outcomes. Handwashing (HW) knowledge has been linked to HW practice in adolescents [9].

Interventions to promote nutrition, hydration, and HL are crucial for protecting children against diseases related to inadequate water, sanitation, and hygiene (WaSH). Examples of such diseases include diarrhoea and helminth infections, which are associated with decreased academic performance and absenteeism [10]. Without effective intervention, these diseases can lead to impaired immune function [11] and chronic diseases, e.g., anemia [12], whose effects may last into adulthood. The social costs of chronic WaSH-related diseases include work absence, decreased wages, and productivity loss [13]. It is known that children who are malnourished and/or dehydrated may be at increased risk for cognitive impairments, which could decrease academic performance and increase school absence and dropout. Furthermore, inadequate WaSH in schools is a contributing factor of children’s malnutrition, especially in the Global South. This work provides new knowledge about a comprehensive school WaSH intervention package that was comprised of policy workshops, health education, provision of hygiene supplies, and installation and repair of WaSH facilities. Specifically, it describes the intervention’s effects on malnutrition, dehydration, HL, and HW. The topic is original, as no known studies have previously examined these outcomes in this setting using mixed methods. Compared with previous studies, our study provides new information about a school WaSH intervention’s effects on dehydration measured by urine specific gravity, as well as hygiene-related HL. These two health outcomes have not yet been well understood in the context of school WaSH.

Most school WaSH interventions have aimed at reducing infectious diseases, though it is uncertain which interventions are effective in reducing malnutrition. HL related to HW has been studied in the elderly [14] and menstruating adolescents [15]. Few studies have assessed hygiene-related HL in schoolchildren. The purpose of our study is to promote children’s health by bridging these knowledge gaps, testing a school WaSH intervention’s effectiveness. Specifically, was the intervention effective in reducing malnutrition and dehydration and increasing HL and HW? We investigated the role of health education (HE) intensity, hypothesizing that high-intensity HE would be more effective than low-intensity HE in improving desired outcomes.

## Methods

### Study Design

The WaSH in Metro Manila Schools study was a cluster-randomized controlled trial (cRCT) conducted in the Philippines’ National Capital Region. We used a parallel group cRCT design with unequal allocation (ratio 1:8.7) of schools to control and intervention groups (CG and IGs) to enable us to implement the intervention efficiently. We hypothesized that the intervention would improve children’s HL, nutrition, hydration, and HW. We adjusted the trial design to measure group-level differences in selected outcomes by including three clusters (cities), each with different numbers of schools and children who were assessed twice, at baseline and endline. No cluster corrections were used for schools, as they were the units for different treatments. Each assessment cycle lasted about one month and was balanced between the CG and IGs to reduce confounding due to seasonal factors.

For the CG, we delivered the “standard of care” consisting of a WaSH policy workshop for teachers and two HE sessions for children. For the IGs, we randomly assigned schools to one of three arms based on the intensity (low, medium, or high) of HE. We also provided policy workshops for teachers, hygiene supplies, and WaSH facilities repairs. Instead of using a double-size CG to increase power, we made the IGs larger than the CG to increase the precision of the intervention comparison. We previously reported our study design and rationale [16].

The study protocol was approved by the Ethics Committees of the University of Bonn, Germany (Number 216/16) (September 28, 2016), and the University of the Philippines, Manila (Number 2017–0113) (February 23, 2017).

### Participants, study sites, and sample size

In Metro Manila, public schools have inadequate WaSH, and WaSH-related diseases are endemic. During a previous cross-sectional study conducted in March - May 2017, we measured diarrhoea and helminth infection rates at 14% and 29.7%, respectively [17]. We selected schools in Manila, Navotas, and Quezon City, cities that are geographically and demographically representative of Metro Manila. Schools were identified based on a complete census of schools managed by the Philippines Department of Education (DepEd). We recruited 15 public schools that previously participated in our cross-sectional study because of existing trust and cooperation with school principals and personnel. These factors facilitated communication and collaboration, which were important to us, as we wanted a long-term working relationship with schools despite limited time and resources. We offered no monetary reimbursement to participants. Instead we gave participants compensation packages comprised of school and hygiene supplies [16].

The outcomes of HAZ and stunting prevalence are the basis of sample size estimation. It assumes a difference of 0.15 HAZ between the IG and CG, not adjusting for repeated measures within clusters, as well as a relative risk (RR) of stunting of 0.7 or smaller, with 10% prevalence in the CG. We assumed a type I error (α) of 0.05, power (1 − β) of 0.8, a one-sided test for a two-sample comparison of means, and a 10% dropout after baseline. Due to limited resources, the CG was not double sized, limiting our ability to account for multiple hypothesis tests. We previously described our sample size estimation [16]. Briefly, to estimate the sample size, we considered the target population to be all the public school children in Metro Manila, where in School Year 2014–2015 a total of 2,059,447 public school children were enrolled [17]. We inflated the sample by 30% and 45% to account for nonresponse and refusal, respectively, and then inflated the sample by another 5% to account for differences in schools’ enrolment sizes. The target sample size was N = 760; we enrolled 756 students at baseline and surveyed 701 students at endline eight months later (retention rate: 93%).

We previously described our multi-stage cluster sampling strategy [16]. First we recruited schools using a list of 15 schools that participated in our cross-sectional study [18]. Schools were eligible if they were public (i.e. managed by the government), had WaSH facilities available for inspection, and had no other on-going WaSH projects. Second we recruited class sections. At each school, we selected one or two class sections to obtain a target sample of ~ 50 students per school. To avoid interrupting classroom instruction, we recruited entire class sections as a whole rather than groups of students from multiple class sections. We did not re-recruit the students who participated in our previous cross-sectional study because a new school year had begun and caused some students to move to different class sections or schools. Third we recruited children. Participants were eligible for our study if they were in grades five, six, seven, or ten; able to complete our questionnaire independently or with minimal assistance; and able to provide relevant health data. We chose these grade levels to ensure children were developmentally mature enough to use and have perceptions about school WaSH facilities and able to actively participate in our intervention activities.

Prior to on-boarding, school principals gave written informed consent “in loco parentis”, i.e., in the place of parents, for children’s participation. We explained to children our study’s purpose and procedures, and stated that participating in our study was voluntary and that all data would be anonymized, confidential, and would not affect their school grades.

### Randomization and masking

We have previously described how we randomly assigned clusters to treatment using Microsoft© Excel’s random number function (simple randomization) [16]. Briefly, in an Excel worksheet, the names of the 13 schools were listed in the first column, wherein one row represented one school. The four IGs (A - D) were listed in the second column, wherein one row represented one IG. (We previously determined how many schools would be allocated to each group.) Schools were ranked and then assigned to groups A, B, C, or D in the third column using Excel’s random number function. IGA was known as the low-intensity health education (LIHE) group, IGB and IGD were known as the medium-intensity health education (MIHE) group, and IGC was known as the high-intensity health education (HIHE) group. The research supervisor and one research assistant (who was not involved in data collection) performed randomization and assigned schools to IGs. The research supervisor and research team enrolled participants. The investigators were unmasked, while all school principals, teachers, personnel, children, and parents, were masked to treatment assignment. It was not possible for participants to know the treatment assignment of nearby schools because any intervention materials distributed to schools did not uniquely identify treatment status.

We completed baseline surveys and then purposively assigned two schools to the CG. One school had a principal who directly asked to participate in our previous cross-sectional study, while the other school was integrated (i.e., it offered kindergarten through grade 12) and was the location of the pilot testing of our survey instruments.

### Procedures

We designed interventions to increase children’s understanding about WaSH and improve health-related behaviours, specifically HW. Our goal was to empower children to be proactive about reducing their exposure to pathogens in the environment, thereby preventing disease and promoting well-being. During formative research, we learned that there was insufficient knowledge about the health benefits of HW and adequate WaSH management. Thus, we developed an intervention strategy to increase knowledge by engaging directly with children and increasing their enthusiasm about HE and capacity for practicing healthy behaviours, as well as creating enabling environments through the provision of necessary equipment and supplies. Our educational materials were based on the existing DepEd curriculum and open educational resources from the U.S. Environmental Protection Agency and the U.S. National Library of Medicine. We report the content of HE sessions in Additional file 1. We used findings from our cross-sectional study and baseline survey, as well as inputs from research assistants (with expertise in the local context), school principals, teachers, and janitors, and we conducted opinion polls with children. To assess whether our intervention would be acceptable and sustainable, we used participatory research and proactively engaged with stakeholders. We confirmed that intervention materials were delivered to study participants at the start of the trial and we made unannounced visits to schools to periodically assess intervention adherence. We implemented the intervention between June 2017 and March 2018 (Additional file 2).

We previously described the four parts of the intervention [16]. Briefly, we provided: WaSH policy workshops for teachers, HE for children, hygiene supplies, and WaSH facilities improvements (Additional file 3).

The research supervisor conducted an in-person eight-hour training workshop for research assistants before conducting baseline school surveys. We previously reported details about training methods, including the time, place, and duration of training, and teaching aids and technologies [16]. Baseline school surveys were conducted according to protocol (Additional file 4). Eight months later, we conducted endline school surveys, using the same methodology and measuring the same outcomes assessed at baseline. We also obtained school administrative data from the DepEd and conducted two cross-sectional surveys: a demographic questionnaire for children to assess household-level risk factors and a water quality study to assess exposures to waterborne pathogens in schools and homes.

Further details about our research procedures, including contents of training workshops, intervention components, and adherence promotion strategies were previously reported [16].

### Outcomes

All trial outcomes were observable, measurable, pre-specified, and assessed at baseline and endline (Additional file 5: Table S1). Trial outcomes were: height-for-age Z score (HAZ), body mass index-for-age Z score (BAZ), body mass index (BMI), height, and weight; prevalence of stunting (HAZ < -2), undernutrition (a composite of thinness [− 3 < BAZ < − 2] and severe thinness [BAZ < − 3]), and overnutrition (a composite of overweight [1 < BAZ < 2] and obesity [BAZ > 2]); urine specific gravity (U_sg_) and prevalence of any (U_sg_ ≥ 1.020), mild (U_sg_ = 1.020), moderate (U_sg_ = 1.025), and severe dehydration (U_sg_ = 1.030) [19]; scores for overall HL, HL about germs, and HL about HW. We calculated HAZ and BAZ using the WHO AnthroPlus (for children 5–19 years old) software (version 3.2.2., WHO, Geneva, Switzerland). We classified nutrition status using the 2007 WHO Growth Reference [20]. During initial trial registration, we erroneously omitted dehydration from our study protocol’s list of outcomes; the study protocol has since been updated. We estimated HL scores via a 20-item questionnaire developed and refined by our research team. We asked children about their knowledge about general hygiene, germs, and handwashing. Examples of questions include: “What are germs?” “True or false: If I have germs, then I can have vomiting or diarrhea.” “How long should I wash my hands with soap and water to get rid of germs?” We previously reported details about the health literacy tool and provided a sample questionnaire [16].

We observed the adequacy of schools’ WaSH facilities, assessing availability, accessibility, cleanliness, and functionality, according to guidelines from the DepEd and the Philippines Department of Health (DOH) [21, 22]. We report data on schools’ WaSH facilities in Additional file 6: Table S2.

We pilot-tested and improved data collection tools before beginning this trial and ensured the safety of participants by adhering to research protocols. No contingency plan was deemed necessary for adverse events as our intervention involved no invasive procedures or provision of medications.

We will report additional (cross-sectional) outcomes (e.g., children’s self-reported health status, satisfaction with schools’ WaSH facilities) and associated risk factors in a forthcoming paper. We report the sample sizes of all surveys conducted during this trial in Additional file 7: Table S3.

### Statistical analysis

The research supervisor conducted data analysis according to a pre-specified data analysis plan. We used intention-to-treat analysis, comparing each IG to the CG. We conducted descriptive analysis, pre- and post-intervention, measuring study participation, demographic characteristics, and outcomes of interest. We reported demographic characteristics and household risk factors, including food insecurity, according to study arm. For each outcome, we reported descriptive results (e.g., percentages, frequencies) for each arm, including the estimated effect size and precision. We measured frequencies and interquartile ranges (IQRs) relevant to homes’ demographic makeup. Data from school inspections were summarized at the school-level by measuring the mean scores of individual facility inspections. We measured prevalence rates using contingency tables with estimates of standard error (SE) and precision.

We assessed socioeconomic status (SES) by performing a factor analysis of variables that indicated the possession of household assets, e.g., computer, cell phone, refrigerator, car. The score of the first factor was then divided into three categories using the k-means procedure. Food security status was derived from a factor analysis of variables indicating access to a secure food source, e.g., enough food is available for all members of the household; eats a variety of food; rarely has asked/begged for food; rarely has gone to sleep feeling hungry. The score of the first factor was then divided into three categories using the k-means procedure.

We used multi-level mixed effects regression models to assess intervention effects. We used two-sided tests for primary outcomes to compare study arms. Paired t-tests were used for continuous variables to calculate the mean height and weight differences. For continuous outcomes, we used multilevel mixed-effects linear regression models to estimate intervention effects with measures of precision, i.e., 95% confidence intervals (CI), and p-values. We used regression models to analyse exposure-response: g = (E[Ai]) = β0 + β1Bi + γCi, where Ai is the primary outcome of interest, g is the appropriate link function (identity for height and weight, logistic for stunting and poor HL), Bi is the continuous exposure of interest, and Ci is the vector of confounders. In linear regression models, intervention effects can be interpreted as the adjusted differences in the mean changes of the desired follow-up outcome between the respective IG and the CG. The model included the respective IG, random intercept for the city, and robust standard errors. We adjusted for the child’s sex, age, and desired outcome at baseline, and the parent/caregiver’s education level and SES. In linear regression models that assessed school-level outcomes, we adjusted for other possible confounders, e.g. attendance in primary school, the school’s MOOE budget, handwashing basin-to-student ratio, and the availability of water in the school restroom.

For binary outcomes, we used multilevel mixed-effects logistic regression and Poisson regression models. In logistic regression models, intervention effects were expressed as the odds ratio (OR) of the prevalence at endline of the desired outcome between the respective IG and CG. The model included the respective IG, random intercept for the city, and robust standard errors. We adjusted for the child’s sex, age, and desired outcome at baseline, and the parent/caregiver’s education level and SES. In logistic regression models that assessed school-level outcomes, we adjusted for other possible confounders, e.g. child’s sex, attendance in primary school, the school’s maintenance and other operating expenses (MOOE) budget, handwashing basin-to-student ratio, availability of water in school restroom. In Poisson regression models, intervention effects can be interpreted as the incidence-rate ratio (IRR) of a desired follow-up outcome between the respective IG and the CG. The model included the respective IG, random intercept for the city, and robust standard errors. We adjusted all models for the children’s age and sex, parent’s education level, and family’s SES, as well as the outcome at baseline, when appropriate. We report details of our models in Additional file 8: Table S4.

To control the effect of confounding we used randomization (i.e. randomly assigning 13 schools to one of four intervention groups) and statistical methods (e.g. logistic regression models that were adjusted for possible confounding variables like age, sex, parental education, and SES.

During our assessment of missing data, if no statistically significant difference was found between children who were missing data and children who were not missing data for key outcomes, we concluded that data were missing at random (MAR), though not missing completely at random (MCAR). MAR means that, within groups defined by the observed data, all data had an equal chance of being missing and that the reason why data were missing is due to a known characteristic of the data themselves [23]. This is a less realistic occurrence in field research. Possible reasons for MAR in our study included: nonresponse or loss to follow-up due to school absence or discontinued school enrolment. We used Stata, version 15 (StataCorp, College Station, Texas, US) for all statistical analyses.

We retroactively registered our trial in the German Clinical Trials Register (DRKS) (05/08/2020; number DRKS00021623). The trial protocol is available on the DRKS’s website: https://www.drks.de/drks_web/.

### Role of the funding source

The study’s funders had no role in the study design, data collection, analysis, interpretation, or writing of this article.

## Results

### Characteristics of study population

**Fig. 1:**
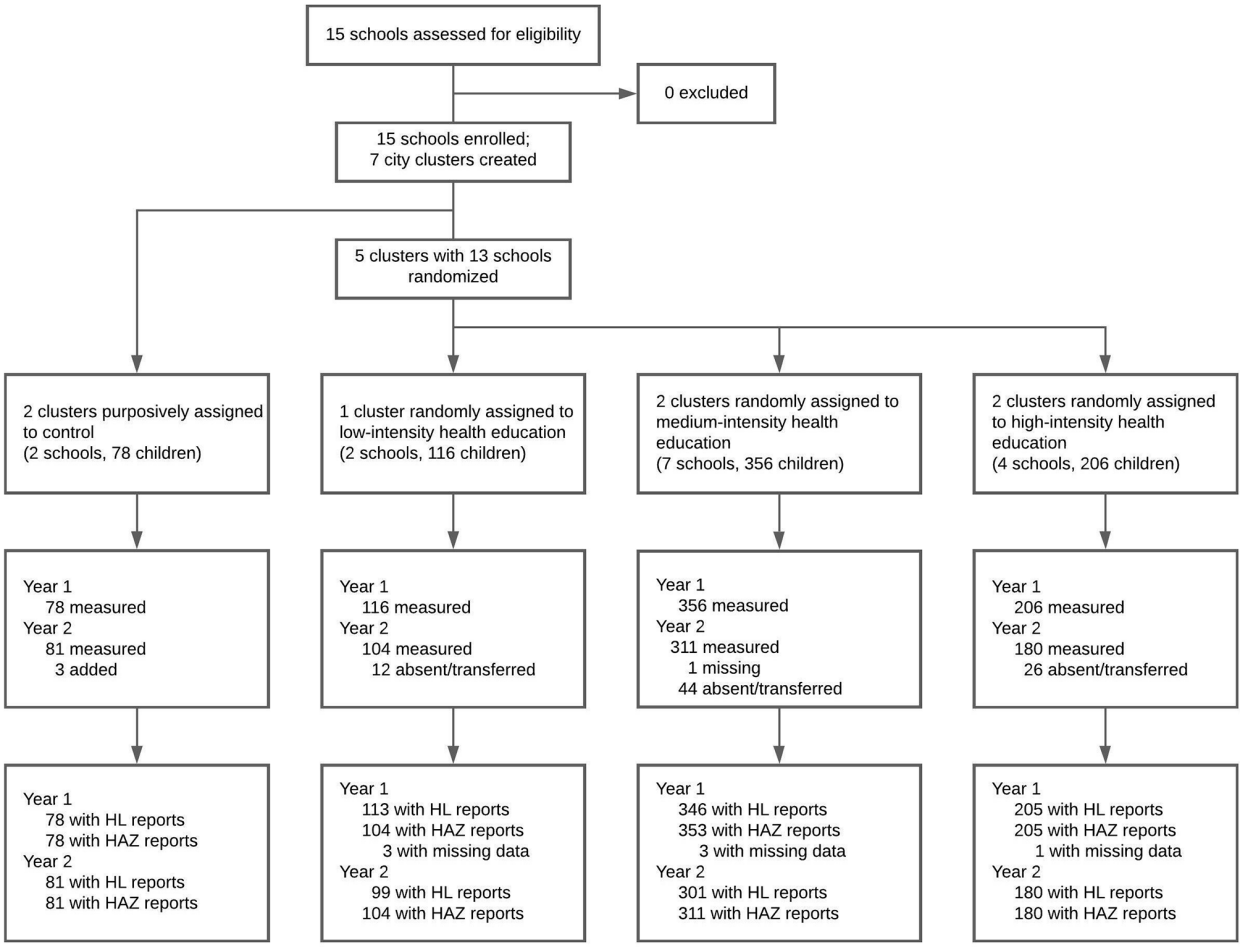
Participant flow diagram. Year 1 refers to baseline (June - August 2017) and Year 2 refers to endline (February - March 2018)

At endline, data on malnutrition and HL were available for 677 and 661 children, respectively. We used the original assignment to study arms, the CG and IGs (LIHE, MIHE, HIHE), during analysis. We based the analysis of intervention effects on the children (n = 596) who provided complete health and HL data during baseline and endline (Fig. 1). From November 2018 to January 2019, 828 children completed the demographic survey; we based our analysis of household-level covariates on this sample of children.

The characteristics of the children who completed the post-intervention demographic study are described in Table 1. At endline, over 25% of the children’s families were of low SES, with about 12%of children reporting that their family shared a toilet with another family and about 8% stating they had no faucet with running water at home. The proportion of children whose parents had earned a college degree differed greatly between the CG (80%) and the IGs (ranging from 47.8 to 57%). During statistical analysis, we accounted for these differences by adjusting for parent’s education level and SES. We report additional covariates in Additional file 9: Table S5.

**Table 1:** Characteristics of study population by study arm

	Control	Low-intensity health education	Medium-intensity health education	High-intensity health education	Total
**Characteristics**	**n (%)**	**n (%)**	**n (%)**	**n (%)**	**n (%)**
*Cluster level (N = 3)*					
Number of schools	2 (13.3)	2 (13.3)	7 (46.7)	4 (26.7)	15 (100)
Number of children	78 (10.3)	116 (15.3)	356 (47.1)	206 (27.2)	756 (100)
Grade range	5, 6	5, 6	5, 6, 7, 10	5, 6, 7	5, 6, 7, 10
School’s age (in years)*, mean (SD)	69.5 (40.3)	34 (15.6)	40.6 (23.1)	51.3 (23)	46.4 (24.5)
School’s annual enrolment*, mean (SD)	2126.5 (1266.4)	6017.5 (3165.7)	4032.1 (2552.2)	4389.8 (2910)	4138.1 (2557.9)
School’s annual MOOE budget (in millions PhP)*, mean (SD)	1.2 (0.9)	3 (1.5)	4 (3.6)	3 (2.2)	3.2 (2.8)
School’s student-to-classroom ratio*, mean (SD)	29 : 1 (12.1)	104 : 1 (19.8)	74 : 1 (18.3)	64.2 : 1 (10.8)	69.4 : 1 (25.1)
School water supply contaminated with coliform (N = 17)	0	2 (50)	2 (28.6)	3 (100)	7 (41.1)
School water supply contaminated with *E.coli* (N = 20)	1 (25)	0	1 (12.5)	1 (25)	3 (15)
*Individual level (N = 756)*					
Female	48 (61.5)	71 (61.2)	198 (55.6)	125 (60.7)	442 (58.5)
Child (age < 13 years)	75 (96.2)	115 (99.1)	261 (73.3)	194 (94.2)	645 (85.3)
*Household factors (N = 828)*	n (%)	n (%)	n (%)	n (%)	n (%)
Demographic and caregiver’s characteristics					
Number of adults in home, median (IQR)	3 (2–4)	3 (2–5)	3 (2–5)	4 (2–6.5)	3 (0–20)
Number of children in home, median (IQR)	3 (2–3)	2 (2–3)	2 (2–4)	3 (2–4)	3 (1–16)
Highest level of education completed by parent/caregiver					
Secondary school and below	17 (20)	48 (42.1)	180 (46.3)	109 (47)	357 (43.1)
Above secondary school	68 (80)	66 (57.9)	209 (53.7)	122 (52.6)	470 (56.8)
*Socioeconomic characteristics (N = 828)*					
Socioeconomic status (SES)					
High	22 (25.9)	1 (0.9)	5 (1.3)	3 (1.3)	31 (3.7)
Middle	58 (68.2)	91 (79.8)	268 (68.9)	161 (69.4)	584 (70.5)
Low	5 (5.9)	22 (19.3)	116 (29.8)	68 (29.3)	213 (25.7)
No refrigerator	8 (9.4)	21 (18.4)	118 (30.3)	74 (31.9)	223 (26.9)
Transportation to school (most common)					
Public	22 (25.9)	2 (1.8)	101 (26)	41 (17.7)	173 (20.9)
Bicycle/pedicab	2 (2.4)	13 (11.4)	26 (6.7)	17 (7.3)	58 (7)
Bus	1 (1.2)	0	2 (0.5)	2 (0.9)	5 (0.6)
Car	26 (30.6)	1 (0.9)	8 (2.1)	6 (2.6)	41 (5)
Jeep	21 (24.7)	2 (1.8)	99 (25.4)	39 (16.8)	168 (20.3)
Tricycle/scooter/motorcycle	16 (18.8)	63 (55.3)	130 (33.4)	67 (28.9)	276 (33.3)
Walk	19 (22.4)	35 (30.7)	124 (31.9)	101 (43.5)	280 (33.8)
*Food insecurity factors (N = 828)*					
Food security					
High	48 (56.5)	67 (58.8)	237 (60.9)	127 (54.7)	485 (58.6)
Medium	37 (43.5)	45 (39.5)	146 (37.5)	99 (42.7)	329 (39.7)
Low	0	2 (1.8)	6 (1.5)	6 (2.6)	14 (1.7)
*WaSH-related factors (N = 828)*					
Home has no restroom	1 (1.2)	0	7 (1.8)	4 (1.7)	12 (1.4)
Home restroom is shared with another family	14 (16.5)	7 (6.1)	41 (10.5)	32 (13.8)	95 (11.5)
Home restroom is located outside of home	4 (4.7)	4 (3.5)	32 (8.2)	23 (9.9)	63 (7.6)
Home restroom has no toilet	1 (1.2)	0	17 (4.4)	12 (5.2)	30 (3.6)
Home has no handwashing area	1 (1.2)	4 (3.5)	26 (6.7)	16 (6.9)	47 (5.7)
Home has no faucet with running water	0	6 (5.3)	37 (9.5)	21 (9.1)	64 (7.7)
Home water supply contaminated with coliform (N = 14)	0	2 (50)	0	4 (100)	6 (42.9)
Home water supply contaminated with *E.coli* (N = 14)	1 (50)	2 (50)	0	1 (25)	4 (28.6)

IQR = interquartile range. MOOE = maintenance and other operating expenses. PhP = Philippine Peso. SES = socioeconomic status. SY = school year. WaSH = water, sanitation, and hygiene. Percentages were calculated from smaller denominators than those shown at the top of the table for all variables because of missing values

*Mean age of school = date of our first visit (30 March 2017) subtracted from school’s date of operation. Mean annual enrolment during SY 2017–2018. Mean MOOE budget during fiscal year 2017. Mean classroom-to-student ratio = enrolment from SY 2016–2017 divided by the number of classrooms in 2016. Data source: Philippines Department of Education

### Change in hygiene-related HL and observed HW

The intervention did not affect mean scores for overall HL, overall knowledge about germs, or overall knowledge about HW (Table 2). After the intervention, the proportions of children who received a “passing” score (≥ 60%) for overall HL, overall knowledge about germs, and overall knowledge about HW increased across all study arms (Table 2). Compared to the CG, MIHE (aIRR 1.10; 95% CI 1.04 to 1.15) had significantly greater knowledge about using HW to prevent infection (Additional file 10: Table S6). Compared to the CG, HIHE (aIRR 1.13; 95% CI 1.07 to 1.20) had significantly greater knowledge about using soap and water to remove germs from hands (Additional file 10: Table S6).

**Table 2:** Effect of intervention on children’s hygiene-related health literacy and observed handwashing

Outcome	Study arm	Baseline		Endline		Effect of intervention (95% CI)	p-value
Binary outcomes*		**n**	**%**	**n**	**%**		
Overall health literacy, passing score**	Control	73	93.6	80	98.8		
	Low-intensity education	106	93.8	99	100	1.00 (0.97, 1.03)	0.97
	Medium-intensity education	307	88.7	287	95.4	1.05 (1.02, 1.08)	p < 0.01
	High-intensity education	189	92.2	173	96.1	1.03 (1.00, 1.06)	0.02
Overall knowledge about germs, passing score	Control	74	94.9	77	95.1		
	Low-intensity education	105	92.9	98	99	1.04 (1.01, 1.07)	0.02
	Medium-intensity education	301	87	288	95.7	1.11 (1.08, 1.13)	p < 0.01
	High-intensity education	184	89.8	174	96.7	1.05 (1.03, 1.08)	p < 0.01
Overall knowledge about handwashing, passing score	Control	63	80.8	74	91.4		
	Low-intensity education	98	86.7	97	98	0.98 (0.81, 1.20)	0.88
	Medium-intensity education	281	81.2	266	88.4	1.02 (0.87, 1.19)	0.81
	High-intensity education	171	83.4	160	88.9	0.98 (0.80, 1.20)	0.88
Washed hands after using toilet/urinal***	Control			18	23.7		
	Low-intensity education			15	25.4	1.02 (0.80, 1.31)	0.84
	Medium-intensity education			15	14.2	3.18 (1.34, 7.55)	p < 0.01
	High-intensity education			40	37.8	3.89 (3.74, 4.05)	p < 0.01
Continuous outcomes****		Mean	±SD	Mean	±SD		
Mean (± SD) overall health literacy score	Control	80.3	13.9	89.1	12.4		
	Low-intensity education	86.6	13.3	95.7	7.6	-0.01 (-0.10, 0.08)	0.77
	Medium-intensity education	79.1	19	87	15.5	0.01 (-0.06, 0.08)	0.79
	High-intensity education	81.8	14.2	90.3	13.2	0.01 (-0.10, 0.11)	0.89
Mean (± SD) overall knowledge about germs score	Control	84.3	13.7	92.7	13.3		
	Low-intensity education	90.5	15.1	97.4	7.2	-0.02 (-0.09, 0.04)	0.49
	Medium-intensity education	83.5	19.8	91.2	14.7	0.02 (-0.04, 0.07)	0.53
	High-intensity education	85	15.8	93.1	12.6	0.01 (-0.04, 0.06)	0.66
Mean (± SD) overall knowledge about handwashing score	Control	73.7	20.1	83.6	16.8		
	Low-intensity education	79.9	17	92.9	15.6	0.02 (-0.14, 0.17)	0.84
	Medium-intensity education	72.3	22.6	80.6	20.8	-0.01 (-0.13, 0.11)	0.86
	High-intensity education	76.7	17.2	86	19.6	-0.005 (-0.30, 0.29)	0.98
Mean (± SD) handwashing practice score*****	Control			71.1	19.7		
	Low-intensity education			49.3	10.3	-0.22 (-0.31, -0.13)	p < 0.01
	Medium-intensity education			50.7	12.8	-0.17 (-0.21, -0.13)	p < 0.01
	High-intensity education			36.5	16.3	-0.51 (-0.58, -0.44)	p < 0.01

CI = confidence interval. SD = standard deviation. The p-value refers to the difference in intervention effect between the respective intervention group (IG) and the control group (CG)

*We used a multilevel mixed-effects Poisson regression model to estimate intervention effects, which can be interpreted as the incidence-rate ratio (IRR) of a desired follow-up outcome between the respective IG and the CG. The model included the respective IG, random intercept for the city, and robust standard errors. We adjusted for the child’s sex, age, and desired outcome at baseline, and the parent/caregiver’s education level and socioeconomic status (SES)

**Passing score ≥ 60%

***We used a multilevel mixed-effects logistic regression model to estimate intervention effects expressed as the odds ratio (OR) of the prevalence at endline of the desired outcome between the respective IG and CG. The model included the respective IG, random intercept for the city, and robust standard errors. We adjusted for the child’s sex, attendance in primary school, the school’s maintenance and other operating expenses (MOOE) budget and handwashing basin-to-student ratio, and the availability of water in the school restroom

****We used a multilevel mixed-effects linear regression model to estimate intervention effects, which can be interpreted as the adjusted differences in the mean changes of the desired follow-up outcome between the respective IG and the CG. The model included the respective IG, random intercept for the city, and robust standard errors. We adjusted for the child’s sex, age, and desired outcome at baseline, and the parent/caregiver’s education level and SES

*****We used a multilevel mixed-effects linear regression model to estimate intervention effects, which can be interpreted as the adjusted differences at endline of the desired outcome between the respective IG and the CG. The model included the respective IG, random intercept for the city, and robust standard errors. We adjusted for the child’s sex, attendance in primary school, the school’s MOOE budget and handwashing basin-to-student ratio, and the availability of water in the school restroom

The intervention negatively affected mean observed HW practice scores across all study arms: LIHE adjusted difference − 0.22; 95% CI -0.31 to 0.13; MIHE adjusted difference − 0.18; 95% CI -0.21 to -0.13; HIHE adjusted difference − 0.51; 95% CI -0.58 to -0.44 (Table 2). The highest HW prevalence rate (37.8%) was seen in HIHE. Significantly greater odds for HW after using the toilet/urinal were seen in MIHE (adjusted odds ratio [aOR] 3.18; 95% CI 1.34 to 7.55) and HIHE (aOR 3.89; 95% CI 3.74 to 4.05).

### Change in malnutrition status

The intervention did not affect BAZ, BMI, HAZ, or undernutrition prevalence. After the intervention, stunting prevalence decreased across all study arms (Table 3). The aOR of stunting was significantly lower in LIHE (aOR 0.95; 95% CI 0.93 to 0.96) compared to the CG. aORs for overnutrition decreased in all IGs. We report additional intervention effects on malnutrition and physical growth in Additional files 11 (Table S7) and 12 (Table S8), respectively.

**Table 3:** Effect of intervention on children’s growth and malnutrition status

Outcome	Study arm	Baseline		Endline		Effect of intervention (95% CI)	p-value
		**Mean**	**±SD**	**Mean**	**±SD**		
Continuous outcomes*							
HAZ	Control	-0.07	1.3	-0.15	1.1		
	Low-intensity education	-0.59	1.4	-0.57	1.3	0.22 (-0.26, 0.70)	0.36
	Medium-intensity education	-0.85	1.1	-0.61	3.3	0.28 (-0.19, 0.75)	0.24
	High-intensity education	-0.75	1.1	-0.82	1	0.21 (-0.17, 0.58)	0.28
BAZ	Control	0.31	1.7	0.64	1.5		
	Low-intensity education	0.24	1.9	0.23	1.5	-0.18 (-0.57, 0.21)	0.37
	Medium-intensity education	-0.15	1.5	-0.12	1.5	-0.06 (-0.38, 0.26)	0.72
	High-intensity education	-0.15	1.4	-0.16	1.4	-0.10 (-0.41, 0.21)	0.53
BMI	Control	18.8	4.5	20.2	4.7		
	Low-intensity education	19	6.9	19.2	4.4	-0.78 (-1.54, -0.01)	0.05
	Medium-intensity education	18.5	3.8	18.9	3.8	-0.28 (-0.95, 0.39)	0.41
	High-intensity education	17.7	3.3	18.1	3.4	-0.33 (-0.94, 0.28)	0.29
Height (cm)	Control	143.5	11.2	147.1	9		
	Low-intensity education	140.3	10.6	145.2	9.6	0.46 (0.30, 0.63)	p < 0.01
	Medium-intensity education	145.5	11.2	149.1	11.2	0.98 (-0.06, 2.02)	0.07
	High-intensity education	140.5	9.3	143.8	8.6	0.57 (0.05, 1.10)	0.03
Weight (kg)	Control	39.2	12.1	44.2	12.7		
	Low-intensity education	37.5	12.6	41.3	13.2	0.08 (-0.17, 0.33)	0.53
	Medium-intensity education	39.8	11.7	42.6	11.8	0.24 (0.07, 0.41)	0.01
	High-intensity education	35.4	9.9	38	10	0.14 (-0.29, 0.57)	0.53
Binary outcomes**		n	%	n	%		
Stunting	Control	4	5.1	4	4.9		
	Low-intensity education	14	13.5	11	10.6	0.95 (0.93, 0.96)	p < 0.01
	Medium-intensity education	47	13.3	41	13.2	1.04 (0.96, 1.13)	0.29
	High-intensity education	24	11.7	19	10.5	1 (0.80, 1.25)	1
Undernutrition	Control	6	7.7	3	3.7		
	Low-intensity education	8	7.7	6	5.8	0.77 (0.09, 6.52)	0.81
	Medium-intensity education	31	8.8	24	7.7	0.93 (0.16, 5.67)	0.94
	High-intensity education	13	6.3	13	7.2	1 (0.19, 5.26)	1
Overnutrition	Control	27	34.6	36	44.4		
	Low-intensity education	32	30.8	31	29.8	0.57 (0.34, 0.96)	0.04
	Medium-intensity education	76	21.5	68	21.9	0.68 (0.37, 1.26)	0.22
	High-intensity education	44	21.4	38	21	0.63 (0.42, 0.93)	0.02

BAZ = body mass index-for-age Z score. BMI = body mass index. CG = control group. CI = confidence interval. cm = centimetre. HAZ = height-for-age Z score. IG = intervention group. kg = kilogram. SD = standard deviation

The p-value refers to the difference in intervention effect between the respective intervention group (IG) and the control group (CG). We classified nutrition status using the 2007 WHO Growth Reference. Stunting = HAZ < − 2 SD. Undernutrition = composite variable comprised of thinness (− 3 < BAZ < − 2) and severe thinness (BAZ < − 3). Overnutrition = composite variable comprised of overweight (1 < BAZ < 2) and obesity (BAZ > 2).

*We used a multilevel mixed-effects linear regression model to estimate intervention effects, which can be interpreted as the adjusted differences in the mean changes of the desired follow-up outcome between the respective IG and the CG. The model included the respective IG, random intercept for the city, and robust standard errors. We adjusted for the child’s sex and age and the parent/caregiver’s education level and socioeconomic status (SES).

**We used a multilevel mixed-effects logistic regression model to estimate intervention effects, which can be expressed as the odds ratio (OR) of change in prevalence of a desired follow-up outcome between the respective IG and the CG. The model included the respective IG, random intercept for the city, and robust standard errors. We adjusted for the child’s sex and age, and the parent/caregiver’s education level and socioeconomic status SES.

### Change in dehydration

After the intervention, aORs and IRRs for dehydration significantly decreased in all IGs: moderate dehydration (LIHE: aOR 0.13; 95% CI 0.08 to 0.22); severe dehydration (MIHE: aIRR 0.02; 95% CI 0.01 to 0.04; HIHE: aIRR 0.01; 95% CI 0.00 to 0.16) (Table 4). We report intervention effects on additional hydration indicators in Additional file 13: Table S9.

**Table 4:** Effect of intervention on children’s dehydration status

Outcome	Study arm	Baseline		Endline		Effect of intervention (95% CI)	p-value
		n	%	n	%		
Severe dehydration*	Control	57	74	63	80.8		
	Low-intensity education	55	53.4	85	81.7	0.84 (0.25, 2.84)**	0.78
	Medium-intensity education	217	64.2	190	57.9	0.02 (0.01, 0.04)**	p < 0.01
	High-intensity education	116	56.6	123	68.3	0.01 (0.00, 0.16)**	p < 0.01
Moderate dehydration	Control	8	10.4	10	12.8		
	Low-intensity education	26	25.2	4	3.9	0.13 (0.08, 0.22)***	p < 0.01
	Medium-intensity education	63	18.6	42	12.8	0.73 (0.32, 1.70)***	0.47
	High-intensity education	42	20.5	23	12.8	0.62 (0.08, 5.04)***	0.66
Mild dehydration	Control	4	5.2	3	3.9		
	Low-intensity education	10	9.7	8	7.7	0.52 (0.02, 16.1)**	0.71
	Medium-intensity education	25	7.4	40	12.2	0.70 (0.04, 11.9)**	0.80
	High-intensity education	26	12.7	14	7.8	0.41 (0.03, 5.87)**	0.51

aIRR = adjusted incidence-rate ratio. aOR = adjusted odds ratio. CG = control group. CI = confidence interval. IG = intervention group. SES = socioeconomic status. U_sg_ = urine specific gravity

The p-value refers to the difference in intervention effect between the respective IG and the CG

*We defined dehydration according to U_sg_. Any dehydration, U_sg_ ≥ 1.020. Mild dehydration, U_sg_ = 1.020. Moderate dehydration, U_sg_ = 1.025. Severe dehydration, U_sg_ = 1.030

**We used a multilevel mixed-effects Poisson regression model to estimate intervention effects, which can be interpreted as the aIRR of a desired follow-up outcome between the respective IG and the CG. The model included the respective IG, random intercept for the city, and robust standard errors. We adjusted for the child’s sex, age, and SES

***We used a multilevel mixed-effects logistic regression model to estimate intervention effects, which can be expressed as the aOR of change in prevalence of a desired follow-up outcome between the respective IG and CG. The model included the respective IG, random intercept for the city, and robust standard errors. We adjusted for the child’s sex, age, and SES

## Discussion

We found no effects of any interventions on mean overall HL scores, BAZ, BMI, HAZ, or prevalence of undernutrition and mild dehydration. There were no intervention effects of any interventions found on schools’ WaSH facilities’ cleanliness, having a dry floor, having no signs of mould or damage in restrooms. No intervention effects were found on water or soap availability in school restrooms.

Meta-analyses and systematic reviews report mixed results about the impact of WaSH interventions on child growth [24–26]. Our trial findings were consistent with trials that reported school WaSH interventions had no significant effects on undernutrition. For example, a trial conducted in schools in Burkina Faso, involving 360 children, aged 8–15 years old, showed that school garden, nutrition, and WASH interventions did not decrease undernutrition prevalence [27]. A trial conducted in schools in Cambodia, Indonesia and Lao People’s Democratic Republic that involved six and seven year-old children (baseline N = 1,847; endline N = 1,499) showed that group tooth brushing and handwashing, combined with deworming and the construction of handwashing units, did not reduce thinness prevalence [28].

While our results are in line with the results of some studies, the results of other studies do not support our results. For example, during our trial, the intervention reduced stunting prevalence. However, a trial conducted in schools in Nepal included 682 children, aged 8–17 years old, and showed that school gardens combined with WaSH interventions did not decrease stunting prevalence [29]. During our trial, the intervention increased observed HW. However, a trial conducted in 10 intervention (average N = 420 students/school) and 10 control (average N = 449 students/school) schools in the Philippines showed that a school WaSH intervention did not improve HW practice [30]. Of note, this trial assessed HW practice via soap use ratio rather than direct observation of HW, which we assessed during our trial.

Yet, comparing trials should be done with caution due to differences in contexts and interventions. Possible reasons for different results reported by previous trials include: diverse intervention components and intervention delivery techniques; varying degrees of adherence to trial protocols, as well as to the intervention. Results from trials that report minimal or mixed intervention effects should not be interpreted as though school WaSH interventions should be abandoned. Rather, it is a reason to pay closer attention to the complexity of school WaSH and develop strategies that address a unique set of challenges with WaSH provision. Haque and Freeman describe these challenges as: “*a*) complex innovation and implementation requirements; *b*) limited external validity of interventions; *c*) inconsistent development sector objectives; and *d*) diverse service providers working at multiple levels” [31].

### Effects on hygiene-related HL and HW

Mean overall HL score increased across all study arms. Of note, baseline overall HL scores were relatively high (mean 90%) to begin with. Perhaps the lack of significant effects was due to the HL questionnaire we developed, as it might have been too easy for children to answer and did not adequately assess their knowledge. Interventions with medium- and high-intensity HE significantly increased children’s odds of receiving a passing score on HL overall. Our findings are consistent with other studies that demonstrated the positive impact of multiple HE sessions on children’s health knowledge [32, 33].

Evidence from a 2018 systematic review showed that adolescents’ HL was associated with health behaviours [34]. Thus, it is important to not ignore the value of HL when designing WaSH interventions. Strong evidence from other systematic reviews shows that children’s/adolescents’ HL is influenced by parents’ education and SES [35, 36]. A greater proportion of children from the CG reported having highly educated parents and higher SES compared to the IGs. Thus, the IGs may have been negatively impacted by having less educated parents and/or lower SES, corroborating results from previous studies [36, 37]. In our study, all IGs had significantly lower mean HW practice scores compared to the CG. Although we adjusted our models for parent’s education and SES, it is possible that the lower scores of the IGs were due to parents’ lower education and lower SES compared to those of the CG. Children whose HW practice was assessed were randomly observed. Thus, some children could have been participants in our survey who received classroom interventions, while other children could have been non-participants who received no classroom interventions.

Medium- and high-intensity HE positively influenced observed HW prevalence, and medium-intensity HE positively increased HW with soap and using the correct HW technique. Yet low- and high-intensity HE negatively influenced children’s use of the correct HW technique. These conflicting findings demonstrate the complexity of modifying health behaviours like HW. Some trials have reported that WaSH interventions increased soap usage but had no effect on HW practice [30, 38].

### Effects on malnutrition status

Our mixed results support findings from meta-analyses and systematic reviews that reported how WaSH interventions may be inadequate in improving children’s nutrition status [24–26]. Based on the results from three large-scale trials, it seems that WaSH alone may be insufficient for reducing child stunting [39].

Although children may not be the primary decision-makers with regards to food choices at home (where parents often buy and prepare meals), they often choose what food to purchase at school and from street vendors. Thus, children must be educated so they can independently make healthy food choices. Previous studies have shown that educating children can help them make nutritious food choices [29].

Stunting and undernutrition are persistent health problems that are likely influenced by many factors, some of which are outside the scope of schools Nevertheless, school WaSH interventions should not be ignored. Recent trials reported that school WaSH interventions could be promising, not only for malnutrition (anaemia) [29], but also for diseases that increase risks for malnutrition like enteric diseases [40].

### Effects on dehydration

Low-intensity HE reduced the prevalence of moderate dehydration but greater HE intensities were needed to reduce severe dehydration. Increasing access to water at schools can increase hydration [41] and decrease dehydration prevalence [42] in children.

During a separate analysis, we compared performance on the HL questionnaire between dehydrated and non-dehydrated children. A significantly greater proportion of children with no dehydration knew when to wash their hands compared to children with mild dehydration. Hydration is important in schoolchildren because dehydration negatively affects cognitive performance [43, 44], which could lead to the practice of unhealthy behaviours.

### Study limitations

We did not randomly assign schools to the CG. Considering the possible significant differences between schools in the CG and IG, and the smaller sample size of the CG, it was possible that no cases of less common outcomes (e.g., thinness, severe thinness) were reported. These null data made it impossible to use regression models to measure the effect of interventions after implementation. Because we did not have a double-sized CG, our ability to account of multiple hypothesis tests was limited. We did not randomly select class sections of children. Rather, school personnel selected the class sections that would be involved in our study. It is possible that the school personnel were biased towards class sections with the best academic performance, including exceptionally gifted children. Thus, the generalizability of our study findings may be limited to similar sub-populations of children, rather than the general population. We created a HL questionnaire rather than use an existing instrument. It is possible that our questionnaire had limited reliability and validity. We assessed dehydration by U_sg_ as measured by urine test strips, which depends on researchers recognizing sometimes subtle colour changes. This method may have limited reliability compared to a urine refractometer. We did not triangulate our measurements with other indicators of dehydration, e.g., physical symptoms like dry lips or subjective complaints of thirst. Because we collected demographic data at endline, we cannot be sure if social factors predisposed children to the health outcomes we measured. However, in this population the social factors were not likely to have changed much between baseline and endline. Due to limited resources, we conducted our trial in urban areas only. Similar studies in rural areas would be of interest as environmental factors and disease transmission pathways may be different in less densely populated settings.

### Practical implications

The success of school WaSH programs depend heavily on intervention implementers (i.e. research assistants) on the one hand, and on intervention beneficiaries on the other hand. We did not assess intervention delivery or adherence in this study. However, some of our findings suggest that intervention implementation likely varied across schools. One reason for variance was a rotating team of different research assistants who implemented the intervention. Although all research assistants received training and delivered the intervention according to protocol, there were differences in their public speaking skills, competence, and enthusiasm–all of which influenced the degree to which they could engage schoolchildren and promote active learning and/or behavior change. Therefore, future intervention studies should take the role of intervention implementers into consideration by providing training and opportunities to practice skills that facilitate engagement with study participants.

Another reason for intervention delivery variance was the presence of different school factors that could not be fully controlled or accounted for, in spite of our research methodology and use of a research protocol that included standardized data collection tools. For example, some schools had very committed school principals who took ownership of WaSH management, while other schools had school principals who were less proactive. Another example is that some teachers had greater capacity to reinforce the WaSH lessons our research team delivered, while other teachers had limited capacity. Thus, future studies should consider the role that school leadership plays in WaSH management by helping school principals and teachers develop a sense of ownership and become empowered to lead improvements, while addressing barrier to change.

During our study we collaborated with multiple stakeholder groups from the education sector, local government, school staff, students’ parents and families, and community partners. Future studies should seek “buy-in” from stakeholders because a sense of ownership could facilitate intervention delivery, adherence, and sustainability. Some strategies for obtaining buy-in include performing outreach to stakeholders and encouraging stakeholder participation.

During our study we assessed conventional health indicators (e.g. nutrition status) that may not change dramatically during a short study period. Relying solely on such indicators could affect how the intervention’s effects would be interpreted (i.e. having little to “no” effect). Thus, it is important to assess intermediary health outcomes (e.g. HL and HW practice) which are more likely to change, even during a short time span. Therefore, future studies should consider using our approach of assessing a combination of conventional health indicators and intermediary health outcomes in order to provide a more nuanced description of intervention effects.

Based on our findings, we recommend that future interventions aimed at increasing children’s HL not only focus on education intensity, but also on health-related contents and the quality of education delivery. Our experience shows the importance of using interactive teaching methods that engage students in discussion and provide opportunities for role playing healthy behaviors. Interactive teaching methods could be more effective in increasing HL compared to traditional lectures that reply only on passive learning. More research is nevertheless needed in light of our study’s limitations. We recommend future studies of longer duration and the use of indicators that directly assess cognitive function (e.g. attention, memory).

### Strengths

By assessing a wide array of indicators, including water quality, household WaSH, and food insecurity, we may help increase the understanding of malnutrition and its causes. Our trial, unlike previous ones, involved varying the intensity of HE and assessing changes in hydration. Our counting of *E. coli* and coliform colonies enabled an unbiased indicator of faecal contaminant exposure. The study design and use of standardized data collection allow study findings to be generalized to populations from similar high-need, low-resource settings in the tropics. Our findings are relevant for efficacy studies that similarly involve intensive intervention promotion.

To our knowledge, we are the first to report that a school WaSH intervention reduced overnutrition. This is important because childhood obesity is a becoming more prevalent in LMICs [1], increasing the risk of NCDs. Despite methodological limitations, our trial provides evidence that comprehensive school WaSH interventions are able to improve direct and indirect determinants of malnutrition, such as hygiene-related HL, HW practice, and dehydration. One advantage of the tested intervention is that it can be easily replicated and quickly and affordably implemented on a larger scale in similar settings. Another advantage is the trial’s potential to promote public health by providing a blueprint for evaluating the impacts of a school WaSH program. In particular, findings from our trial can be targeted to help vulnerable populations such as urban poor children and adolescents living in a tropical megacity in the Global South, where a lack of data about health, nutrition, and environmental exposures continues to hamper public health efforts. Comprehensive school WaSH could improve children’s HL and HW, thereby reducing malnutrition and dehydration. In order for benefits to be maintained over the lifespan, school WaSH may likely need to be linked to parent/caregiver involvement and household WaSH.

In spite of our study’s limitations, we have provided findings that contribute to the understanding of health promotion from a theoretical perspective. Specifically, our work extends and refines the Social Ecological Model (SEM) which (1) describes how behavior is influenced by the social environment and vice versa; and (2) includes different levels of influence, e.g. individual, classroom, and school [45, 46]. The SEM stipulates that people find it easier to adopt healthy behaviors when their environment is conducive to change. Thus, we implemented a multicomponent intervention package that reached individuals, classrooms, and school staff in order to improve WaSH environments in a way that facilitated proper toilet use and HW. For example, HW is influenced by self-efficacy at the individual level, social support from peers at the classroom level, and perceptions of cleanliness at the school level. Because the SEM suggests that multiple levels of influence interact with each other, we widened our focus beyond interventions aimed only at the individual. We also addressed the classroom and school levels by targeting policies and the built environment.

## Conclusion

Our trial prompts further investigation about comprehensive, yet context-specific, school WaSH interventions, examining their ability to prevent malnutrition and promote HW practice. Our findings suggest that medium- and high-intensity HE may not be more effective than low-intensity HE in improving growth or HW, and that comprehensive school WaSH interventions can reduce but may not eliminate stunting. Our trial suggests that a holistic approach to managing school WaSH, when coupled with supplementary HE aimed at engaging and empowering children, has the potential to increase children’s awareness and practice of hygiene behaviours, thereby reducing infectious diseases and improving nutrition status. We recommend incorporating developmentally appropriate HE strategies (e.g., poster-making, restroom-cleaning contests, role playing, song-writing workshops) and enhancing existing school WaSH programs that promote policy enforcement, the provision of hygiene supplies, and maintenance of WaSH facilities. HE strategies targeted specifically at older children and adolescents, and outreach to parents/caregivers, are crucial for improving HL and HW and reducing malnutrition and dehydration.

### Electronic supplementary material

Below is the link to the electronic supplementary material.

Supplementary Material 1

## Data Availability

The datasets supporting the conclusions of this article are available upon request made with the corresponding author. Datasets will be made available in the Center for Development Research (ZEF), University of Bonn, Data Portal website, [https://daten.zef.de/geonetwork/srv/eng/catalog.search#/home]. The study protocol and data collection tools are available (open access) in the public domain at 10.3390/ijerph18010226. Analytic codes are included in the Supplementary Information of this article.

## Abbreviations

aIRR: adjusted incidence-rate ratio
aOR: adjusted odds ratio
BAZ: body mass index-for-age Z-score
BMI: body mass index
CG: control group
CI: confidence interval
cm: centimeter
cRCT: cluster-randomised controlled trial
DepEd: Philippines Department of Education
DOH: Philippines Department of Health
DRKS: Deutschen Register Klinischer Studien (German Clinical Trials Register)
*E.coli*: *Escherichia coli*
EPA: United States Environmental Protection Agency
GDP: gross domestic product
HAZ: height-for-age Z-score
HE: health education
HIHE: high-intensity health education
HL: health literacy
HW: handwashing
IG: intervention group
Inc.: incorporation (business)
IQR: interquartile range
kg: kilogram
LIHE: low-intensity health education
LMIC: low- and middle-income country
Ltd.: limited (company)
MAR: missing at random
MCAR: missing completely at random
MIHE: medium-intensity health education
MOOE: Maintenance and Other Operation Expenses
NCD: non-communicable disease
OR: odds ratio
PhP: Philippine Peso
PRC: People’s Republic of China
PTA: Parent-Teacher Association
RR: relative risk
SD: standard deviation
SE: standard error
SES: socioeconomic status
SY: school year
U_sg_: urine specific gravity
U.S.A.: United States of America
USD: United States Dollar
UN: United Nations
WaSH: water, sanitation, and hygiene
WHO: World Health Organization
