# Correction: School water, sanitation, and hygiene (WaSH) intervention to improve malnutrition, dehydration, health literacy, and handwashing: a cluster-randomised controlled trial in Metro Manila, Philippines

**DOI:** 10.1186/s12889-023-17425-6

**Published:** 2024-04-10

**Authors:** Stephanie O. Sangalangen, Allen Lemuel G. Lemence, Zheina J. Ottong, John Cedrick Valencia, Mikaela Olaguera, Rovin James F. Canja, Shyrill Mae F. Mariano, Nelissa O. Prado, Roezel Mari Z. Ocaña, Patricia Andrea A. Singson, Ma. Lourdes Cumagun, Janine Liao, Maria Vianca Jasmin C. Anglo, Christian Borgemeister, Thomas Kistemann

**Affiliations:** 1 Center for Development Research, University of Bonn, Genscherallee 3, 53113 Bonn, Germany; 2 Department of Industrial Engineering, University of the Philippines Los Baños, Los Baños, Philippines; 3 School of Earth Sciences and Environmental Engineering, Gwangju Institute of Science and Technology, Gwangju, South Korea; 4 National Institute of Physics, College of Science, University of the Philippines Diliman, Quezon City, Philippines; 5 Philippines Department of Education, Meralco Avenue, Pasig City, Philippines; 6 College of Mass Communication, University of the Philippines Diliman, Quezon City, Philippines; 7 Marine Science Institute, University of the Philippines Diliman, Quezon City, Philippines; 8 Department of Environment Systems, University of Tokyo, Kashiwa, Chiba Japan; 9 National Institute of Geological Sciences, University of the Philippines Diliman, Quezon City, Philippines; 10 School of Medicine, Far Eastern University - Nicanor Reyes Medical Foundation, Quezon City, Philippines; 11 School of Social Sciences, Ateneo de Manila University, Quezon City, Philippines; 12 Department of Science and Technology, Food and Nutrition Research Institute, Taguig, Philippines; 13 School of Diplomacy and Governance, De La Salle - College of Saint Benilde, Manila, Philippines; 14 Department of Psychology, University of the Philippines Diliman, Quezon City, Philippines; 15 Institute of Hygiene and Public Health, University of Bonn, Bonn, Germany

BMC Public Health (2022) 22:2034

10.1186/s12889-022-14398-w

The original publication of this article contained 2 errors. The incorrect and correct information is listed below. The original article has been updated.

Incorrect author name & affiliation

Maria Lourdes Cumagun, Department of Science and Technology, Nutrition Research Institute, Taguig, Philippines

Correct author name & affiliation

Ma. Lourdes Cumagun, Department of Science and Technology, Food and Nutrition Research Institute, Taguig, Philippines

