# Supplementary material for: School water, sanitation, and hygiene (WaSH) intervention to improve malnutrition, dehydration, health literacy, and handwashing: a cluster-randomised controlled trial in Metro Manila, Philippines
Source: BMC Public Health. 2022 Nov 7;22:2034. doi: 10.1186/s12889-022-14398-w (PMC9641834; doi:10.1186/s12889-022-14398-w)
Supplement: Supplementary file 1 — Supplementary Material 1 [file 12889_2022_14398_MOESM1_ESM.docx]

**School water, sanitation, and hygiene (WaSH) intervention to improve malnutrition, dehydration, health literacy, and handwashing: a cluster-randomized controlled trial in Metro Manila, Philippines**

Stephanie O Sangalang, Allen Lemuel G Lemence, Zheina J Ottong, John Cedrick Valencia, Mikaela Olaguera, Rovin James F Canja, Shyrill Mae F Mariano, Nelissa O Prado, Roezel Mari Z Ocaña, Patricia Andrea A Singson, Maria Lourdes Cumagun, Janine Liao, Maria Vianca Jasmin C Anglo, Christian Borgemeister, Thomas Kistemann

Correspondence: Stephanie O. Sangalang, Center for Development Research, University of Bonn, Germany. **ssangala@uni-bonn.de**

**Supplementary Information**

**Table of contents**

| **Additional file 1** | Health education session content by intervention arm. |
| --- | --- |
| **Additional file 2.** | Project timeline. |
| **Additional file 3.** | Summary of school WaSH intervention components. |
| **Additional file 4.** | Summary of conduct of baseline school survey. |
| **Additional file 5: Table S1.** | Trial outcomes, expected results, contents of health education intervention, and training provided to research assistants. |
| **Additional file 6: Table S2.** | Effect of intervention on the adequacy of schools’ WaSH facilities. |
| **Additional file 7: Table S3.** | Surveys conducted during this trial: outcomes, covariates, and sample sizes. |
| **Additional file 8: Table S4.** | Regression models used to measure intervention effects. |
| **Additional file 9: Table S5.** | Expanded table: characteristics of study population by study arm. |
| **Additional file 10: Table S6.** | Expanded table: effect of intervention on children’s hygiene-related health literacy and observed handwashing. |
| **Additional file 11: Table S7.** | Expanded table: effect of intervention on children’s malnutrition status. |
| **Additional file 12: Table S8.** | Effect of the intervention on changes in children’s growth indicators. |
| **Additional file 13: Table S9.** | Expanded table: effect of intervention on children’s dehydration status and urine specific gravity. |
| See attached file. | CONSORT 2010 checklist for reporting a cluster-randomized controlled trial. |

| **Study arm** | **Session number** | | | |
| --- | --- | --- | --- | --- |
|  | **1** | **2** | **3** | **4** |
| Control | X |  |  | X |
| LIHE | X |  |  | X |
| MIHE | X | X |  | X |
| HIHE | X | X | X | X |

| **1. What are germs?**  1.1 What are germs?  1.1.1 Symptoms of infection  1.2 What do germs look like?  1.2.1 Bacteria: *E. coli*  1.2.2 Virus: influenza  1.2.3 Fungi: *Candida albicans*  1.3 Where can germs be found?  1.3.1 Human body  1.3.2 Food & water  1.3.3 Animals  1.3.4 Environment: school restroom  1.4 What can get rid of germs?  1.5 What are the five steps of proper handwashing? | **2. What is environmental health?**  2.1 Elements of environment  2.2 Importance of water  2.3 Bodies of water in the Philippines  2.4 Water cycle  2.5 Water pollution  2.6 Water & health  2.7 Consequences of unclean water |
| --- | --- |
| **3. Climate safety**  3.1 Safe sun exposure  3.1.1 What is the sun?  3.1.2 Three things that the sun gives us  3.1.3 Too much sun exposure  3.1.4 Sun protection  3.1.5 Dangers of skin “whitening”  3.2 Natural disasters preparedness  3.2.1 Types of natural disasters  3.2.2 Common natural disasters in the Philippines  3.2.3 Why the Philippines is at high risk  3.2.4 How to survive  3.3 What is climate change? | **4. Health promotion**  4.1 What does “health” mean?  4.2 Healthy habits  4.2.1 Hygiene  4.2.2 Exercise  4.2.3 Sleep  4.2.4 Screen time  4.3 Nutrition  4.3.1 Food pyramid  4.3.2 Balanced diet  4.3.3 Which dish should you eat?  4.3.4 Malnutrition  4.4 Disease prevention  4.4.1 Vaccines  4.4.2 Avoid stress  4.4.3 Adequate WaSH |

HIEH = high-intensity health education. LIHE = low-intensity health education. MIHE = moderate-intensity health education.

**Additional file 1:** Health education session content by study arm.

Q = quarter. WaSH = water, sanitation, and hygiene.

Formative research: pilot testing of data collection tools; cross-sectional study about association between school WaSH and children’s diarrhoea, helminth infection, malnutrition, dehydration (N = 1,558); cross-sectional study about household WaSH and parents’ perceptions about WaSH and experience with food insecurity.

Survey of schools: data from school principals via interview and from school WaSH facilities via inspection.

Survey of children: data from children via questionnaire and observation.

Training: different research teams (comprised of 4 - 8 investigators) were hired and trained for nearly all phases of the project. The only constant was the research supervisor (i.e. the corresponding author). We have reported the contents of our training modules previously.^16^

Intervention development and implementation: Please refer to the Supplementary Materials of our study protocol.^16^

Hand-over: provision of printed research portfolio to school principal. Portfolio included summary of research activities, results, and recommendations. Provision of certificate of participation to school principal and token of appreciation for study participants. We provided no monetary reimbursement; we reported compensation for study participants previously.^16^

**Additional file 2:** Project timeline.

DepEd = Philippines Department of Education. HE = health education. HIHE = high-intensity health education. HL = health literacy. HW = handwashing. LIHE = low-intensity health education. MIHE = medium-intensity health education. SDG = Sustainable Development Goal. UN = United Nations. WaSH = water, sanitation, and hygiene.

^1^We provided school principals a printed research portfolio with recommendations for enforcing the DepEd’s existing WaSH policies. We conducted one-hour hygiene promotion workshops for teachers to demonstrate the need to improve WaSH in schools, in line with the UN’s SDG 6, related to WaSH, and to comply with the DepEd’s Order No. 10, related to WaSH in schools.

^2^We created a HE package based on HL predictors, facilitators, and barriers. Research assistants delivered between two and four one-hour HE sessions to children based on treatment assignment. Participants in the LIHE group received two sessions, while those in the MIHE and HIEH groups received three and four sessions, respectively. Each HE session comprised of an interactive verbal presentation accompanied by Microsoft© PowerPoint slides, class discussion, and trivia game. To increase children’s interest in WaSH, we used mixed methods as described in our study protocol.^16^

^3^“Hygiene Heroes”, is a Tagalog (Filipino) language educational video about the importance of proper HW. It was developed by the research team and features research assistants in all acting roles. A copy of the video is available upon request from the corresponding author.

^4^Cleaning supplies, e.g. brooms, mops, dustpans, all-purpose cleaner, sponges, wash cloths.

^5^Hygiene supplies, e.g. liquid and bar hand soap, wall-mounted dispensers for liquid soap, menstruation pads, shelving (for storing supplies).

^6^Drinking water with ceramic filter donated by the Philippines Department of Science and Technology. Drinking water with gravity driven membrane donated by the Gwangju Institute of Science and Technology.

**Additional file 3:** Summary of school WaSH intervention components.

6. Handwashing Observation^1^

7.

School

WaSH inspection^1^

| Step | Station | Activities |
| --- | --- | --- |
| 1 | Introduction | After entering the classroom, research assistants introduced themselves to the teacher and children. They explained, in Tagalog (Filipino) language, the study’s purpose, objectives, and procedures. |
| 2 | Registration | Children were assigned a unique study ID number and given a set of 3 tickets labelled with their study ID number. The three tickets corresponded to three research stations: 1) HL questionnaire, 2) urine testing, and 3) height and weight measurement. Children were instructed to give their tickets to the appropriate research assistant at each station after providing the relevant data. |
| 3 | Questionnaire | Children completed the HL questionnaire using the QuickTapSurvey© app (Formstack LLC, Fishers, Indiana, U.S.A.) installed on password-protected tablets and smartphones. Children answered the questionnaire independently and privately, away from the view of their classmates. Research assistants verified the completion of each child’s questionnaire . |
| 4 | a. Height and weight | Research assistants measured children’s standing height (to the nearest cm), without shoes, using a tape measure attached to the wall of the school building. We measured children’s weight (to the nearest 0.1 kg), without shoes or any items inside their pockets, using a digital weighing scale (EKS Asia Ltd., Hong Kong, Special Administrative Region of the People's Republic of China). |
|  | b. Urine | Research assistants asked children to put their freshly collected urine specimen inside a plastic cup that was labelled with their study ID number. Point-of-care urinalysis was performed per protocol with urine test strips (Insight Urinalysis Reagent Strips, Acon Laboratories Inc., San Diego, California, U.S.A.). We interpreted the urinalysis results by comparing the colour changes displayed on the urine test strip to the manufacturer-provided urinalysis interpretation guide. |
| 5 | Quality control | At the end of the school survey, but prior to leaving the school campus, we counted the number of responses saved in the tablets and smartphones. We verified that this number matched the total number of tickets collected from all three research stations (HL questionnaire, height and weight, and urine stations). |
| 6 | Handwashing observation | We conducted unannounced handwashing observations to see if children washed their hands after using the toilet or urinal, and if they used the correct handwashing technique and for the correct duration of time. |
| 7 | School WaSH inspection | We completed school restroom inspection checklists and interviewed school principals or representatives about school WaSH policies and perceptions about WaSH. |

cm = centimetre. ID = identification. kg = kilogram. LLC = limited liability company. Ltd. = limited company. U.S.A. = United States of America. WaSH = water, sanitation, and hygiene.

^1^Steps 6 and 7 are not included in the main process diagram to indicate that they were not necessarily completed during same school visit as steps 1−5.

**Additional file 4:** Summary of conduct of baseline school survey.

| Outcome | Description of outcome | Expected results | Contents of health education intervention | Training provided |
| --- | --- | --- | --- | --- |
| Outcome 1 (Primary outcome) | Change in children’s knowledge about WaSH (e.g., handwashing, germs, infection prevention) | Children will know:   - when to wash their hands. - how long to wash their hands. - how to prevent infection by washing their hands. - that germs can be removed by washing hands with soap and water. - symptoms of infection.   Improvement in health literacy scores specific to germs, infection prevention, and handwashing, among children in the high-intensity HE arm. | **1. What are germs?**  **1.1 What are germs?**  **1.1.1 Symptoms of infection**  1.2 What do germs look like?  1.2.1 Bacteria: E. coli  1.2.2 Virus: influenza  1.2.3 Fungi: Candida albicans  **1.3 Where can germs be found?**  **1.3.1 Human body**  **1.3.2 Food & water**  **1.3.3 Animals**  **1.3.4 Environment: school restroom**  **1.4 What can get rid of germs?**  **1.5 What are the five steps of proper handwashing?** | Lecture, demonstration, small group work, practice with public speaking, child-friendly communication, and classroom management |
| Outcome 2 (Primary outcome) | Change in nutritional status | Improvement in BAZ and HAZ and decreased prevalence of stunting, undernutrition, and overnutrition among children in the high-intensity HE arm. | 4. Health promotion  4.1 What does “health” mean?  4.2 Healthy habits  4.2.1 Hygiene  4.2.2 Exercise  4.2.3 Sleep  4.2.4 Screen time  **4.3 Nutrition**  **4.3.1 Food pyramid**  **4.3.2 Balanced diet**  **4.3.3 Which dish should you eat?**  **4.3.4 Malnutrition**  4.4 Disease prevention  4.4.1 Vaccines  4.4.2 Avoid stress  4.4.3 Adequate WaSH | Lecture, small group work, practice with public speaking, child-friendly communication, and classroom management |
| Outcome 3 | Change in hydration status | Improvement in urine specific gravity among children in the high-intensity HE arm. | 2. What is environmental health?  2.1 Elements of environment  **2.2 Importance of water**  2.3 Bodies of water in the Philippines  2.4 Water cycle  2.5 Water pollution  **2.6 Water & health**  2.7 Consequences of unclean water | Lecture, small group work, practice with public speaking, child-friendly communication, and classroom management |
| Outcome 4 | Change in handwashing practice | Improvement of observed handwashing prevalence in intervention arms. | 1. What are germs?  1.1 What are germs?  1.1.1 Symptoms of infection  1.2 What do germs look like?  1.2.1 Bacteria: E. coli  1.2.2 Virus: influenza  1.2.3 Fungi: Candida albicans  1.3 Where can germs be found?  1.3.1 Human body  1.3.2 Food & water  1.3.3 Animals  1.3.4 Environment: school restroom  **1.4 What can get rid of germs?**  **1.5 What are the five steps of proper handwashing?** | Lecture, demonstration, small group work, practice with public speaking, child-friendly communication, and classroom management |
| Outcome 5 | Change in schools’ WaSH adequacy | Improvement of observed adequacy of WaSH conditions among schools in intervention arms. | N/A | N/A |

BAZ = body mass index-for-age Z score. HAZ = height-for-age Z score. HE = health education. WaSH = water, sanitation, and hygiene. Bold indicates special emphasis given to this topic during health education intervention(s). Additional information can be found in the Supplemental Material Sangalang et al., 2021.

**Additional file 5: Table S1** Trial outcomes, expected results, contents of health education intervention, and training provided to research assistants.

| **Outcome*** | **Study arm** | **Baseline mean +- SD** | **Endline mean +- SD** | **Effect of intervention  (95% CI)** | **p-value** |
| --- | --- | --- | --- | --- | --- |
| **Restroom is clean** | Control | 0.5 (0.71) | 0.39 (0.15) |  |  |
|  | Low-intensity education | 0.29 (0.06) | 0 (-) | -0.23 (-1, 0.54) | 0.57 |
|  | Medium-intensity education | 0.25 (0.39) | 0.33 (0.27) | 0.16 (-0.61, 0.93) | 0.69 |
|  | High-intensity education | 0.15 (0.18) | 0.19 (0.14) | 0.15 (-0.47, 0.77) | 0.64 |
| **Restroom has water available** | Control | 0.75 (0.35) | 0.93 (0.10) |  |  |
|  | Low-intensity education | 0.69 (0.44) | 1 (-) | -0.18 (-0.53, 0.17) | 0.32 |
|  | Medium-intensity education | 0.70 (0.19) | 0.90 (0.19) | 0.02 (-0.26, 0.29) | 0.90 |
|  | High-intensity education | 0.71 (0.21) | 0.89 (0.21) | 0.01 (-0.31, 0.33) | 0.97 |
| **Restroom floor is dry** | Control | 0.50 (0.71) | 0.31 (0.44) |  |  |
|  | Low-intensity education | 0.13 (0.18) | 0 (-) | 0.19 (-0.18, 0.55) | 0.32 |
|  | Medium-intensity education | 0.12 (0.14) | 0.08 (0.17) | 0.15 (-0.14, 0.44) | 0.31 |
|  | High-intensity education | 0.07 (0.13) | 0.08 (0.15) | 0.20 (-0.13, 0.53) | 0.24 |
| **Restroom has no signs of mould** | Control | 0.50 (0.71) | 0.43 (0.61) |  |  |
|  | Low-intensity education | 0.06 (0.09) | 1 (-) | 1.07 (-0.75, 2.89) | 0.25 |
|  | Medium-intensity education | 0.23 (0.27) | 0.71 (0.39) | 0.53 (-0.97, 2.03) | 0.49 |
|  | High-intensity education | 0.61 (0.28) | 0.27 (0.38) | -0.27 (-1.79, 1.25) | 0.73 |
| **Restroom has no flies** | Control | 0.92 (0.12) | 1 (0) |  |  |
|  | Low-intensity education | 0.77 (0.15) | 1 (-) | 0.25 (0.09, 0.41) | p < 0.01 |
|  | Medium-intensity education | 1 (0) | 0.75 (0.41) | -0.35 (-0.90, 0.19) | 0.20 |
|  | High-intensity education | 1 (0) | 0.87 (0.25) | -0.21 (-0.51, 0.08) | 0.16 |
| **All toilets flush** | Control | 0.50 (0.71) | 0.80 (0.08) |  |  |
|  | Low-intensity education | 0.52 (0.21) | 0 (-) | -0.97 (-2.06, 0.11) | 0.08 |
|  | Medium-intensity education | 0.17 (0.25) | 0.55 (0.45) | 0.03 (-0.79, 0.85) | 0.94 |
|  | High-intensity education | 0.17 (0.24) | 0.50 (0.40) | 0.03 (-0.79, 0.85) | 0.94 |
| **Restroom has waste disposal bin available** | Control | 0.52 (0.50) | 0.59 (0.23) |  |  |
|  | Low-intensity education | 0.06 (0.09) | 0.50 (-) | 0.43 (0.05, 0.81) | 0.03 |
|  | Medium-intensity education | 0.41 (0.38) | 0.36 (0.38) | -0.13 (-0.44, 0.17) | 0.40 |
|  | High-intensity education | 0.15 (0.18) | 0.12 (0.19) | -0.09 (-0.45, 0.26) | 0.61 |
| **Restroom has no graffiti** | Control | 0.33 (0.24) | 0.81 (0.27) |  |  |
|  | Low-intensity education | 0.13 (0.18) | 0 (-) | -0.48 (-1.17, 0.21) | 0.18 |
|  | Medium-intensity education | 0.31 (0.28) | 0.68 (0.28) | -0.10 (-0.63, 0.43) | 0.72 |
|  | High-intensity education | 0.58 (0.39) | 0.58 (0.15) | -0.48 (-1, 0.05) | 0.08 |
| **Restroom has no signs of damage** | Control | 0.13 (0.18) | 0.20 (0.08) |  |  |
|  | Low-intensity education | 0 (0) | 0 (-) | -0.07 (-0.21, 0.07) | 0.32 |
|  | Medium-intensity education | 0 (0) | 0.15 (0.15) | 0.06 (-0.06, 0.18) | 0.32 |
|  | High-intensity education | 0.03 (0.07) | 0.12 (0.14) | 0.01 (-0.10, 0.12) | 0.85 |
| **Restroom has door locks available** | Control | 0.58 (0.59) | 0.57 (0.61) |  |  |
|  | Low-intensity education | 0.13 (0.18) | 0 (-) | 0.01 (-0.01, 0.04) | 0.32 |
|  | Medium-intensity education | 0.19 (0.33) | 0.32 (0.40) | 0.18 (-0.09, 0.45) | 0.19 |
|  | High-intensity education | 0.33 (0.31) | 0.28 (0.28) | -0.04 (-0.76, 0.69) | 0.92 |
| **Soap available in restroom** | Control | 0.44 (0.62) | 0.38 (0.53) |  |  |
|  | Low-intensity education | 0 (0) | 0 (-) | 0.06 (-0.06, 0.18) | 0.32 |
|  | Medium-intensity education | 0.07 (0.13) | 0.15 (0.25) | 0.10 (-0.09, 0.29) | 0.29 |
|  | High-intensity education | 0.12 (0.16) | 0.18 (0.23) | 0.13 (-0.05, 0.30) | 0.16 |
| **Lights are functional in restroom** | Control | 0.60 (0.38) | 0.79 (0.30) |  |  |
|  | Low-intensity education | 0.65 (0.03) | 1 (-) | 0.15 (0.04, 0.26) | 0.01 |
|  | Medium-intensity education | 0.50 (0.42) | 0.63 (0.32) | -0.02 (-0.53, 0.50) | 0.95 |
|  | High-intensity education | 0.87 (0.16) | 0.54 (0.44) | -0.51 (-1.08, 0.07) | 0.08 |
| **Restroom is well-lit** | Control | 0.56 (0.09) | 0.79 (0.30) |  |  |
|  | Low-intensity education | 0.77 (0.15) | 0 (-) | -0.89 (-1.19, -0.59) | p < 0.01 |
|  | Medium-intensity education | 0.60 (0.47) | 0.70 (0.36) | -0.11 (-0.81, 0.58) | 0.75 |
|  | High-intensity education | 0.88 (0.25) | 0.84 (0.20) | -0.26 (-0.61, 0.09) | 0.15 |
| **School principal is satisfied with restroom's sanitation**** | Control | 0.50 (0.71) | 0.50 (0.71) |  |  |
|  | Low-intensity education | 0.50 (0.71) | 0 (-) | -- |  |
|  | Medium-intensity education | 0.57 (0.53) | 0.40 (0.55) | 0.76 (0.48, 1.21) | 0.24 |
|  | High-intensity education | 0.50 (0.58) | 0.50 (0.71) | 0.78 (0.59, 1.04) | 0.09 |
| **School principal is satisfied with children's hygiene**** | Control | 0.50 (0.71) | 0.50 (0.71) |  |  |
|  | Low-intensity education | 1 (0) | 1 (-) | 0.74 (0.46, 1.21) | 0.23 |
|  | Medium-intensity education | 0.43 (0.53) | 0.20 (0.45) | 0.57 (0.49, 0.66) | p < 0.01 |
|  | High-intensity education | 0.50 (0.58) | 0.50 (0.71) | 0.78 (0.59, 1.04) | 0.09 |

CG = control group. CI = confidence interval. IG = intervention group. IRR = incidence-rate ratio. MOOE = maintenance and other operating expenses. SD = standard deviation. WaSH = water, sanitation, and hygiene.

The p-value refers to the difference in intervention effect between the respective IG and the CG.

*We used a multilevel mixed-effects linear regression model to estimate intervention effects, which can be interpreted as the adjusted differences in the mean changes of the desired outcome between the respective IG and the CG. The model included the respective IG, random intercept for the city, and robust standard errors. We adjusted for the school’s age, enrolment size, and MOOE budget; adjustments were based on covariates’ measurements at baseline.

**We used a multilevel mixed-effects Poisson regression model to estimate intervention effects, which can be interpreted as the IRR of a desired follow-up outcome between the respective IG and the CG. The model included the respective IG, while adjusting for the school’s enrolment size and clustering by city.

**Additional file 6: Table S2** Effect of intervention on the adequacy of schools’ WaSH facilities.

| Survey | Outcomes | Covariates | Baseline N | Endline N |
| --- | --- | --- | --- | --- |
| Children’s HL questionnaire | Binary variables: HL overall passing score, overall knowledge about germs passing score, overall knowledge about HW passing score | age, sex, grade | 729 | 660 |
|  | Continuous variables: HL overall score, overall knowledge about germs score, overall knowledge about HW score | same as above | same as above | same as above |
| Children’s health examination | Binary variables: stunting, undernutrition, overnutrition, dehydration (mild, moderate, severe, any) | age, sex, grade | Height: 741; weight: 739; urine: 724. | Height: 712; weight: 710; urine: 706. |
|  | Continuous variables: BAZ, HAZ, urine specific gravity | age, sex, grade | same as above | same as above |
| Children’s demographic survey | Binary variables: is healthy; rarely goes to bed while feeling hungry; is satisfied with school restroom; is satisfied with school HW area. | age, sex, grade, parent’s education, parent’s employment, exposure to SHS, home WaSH^1^, SES^2^, number of adults, number of children, parent is illiterate or disabled, food security^3^, school WaSH^4^ | n/a | 828 |
|  |  | Categorical/ordinal variables: source of drinking water, flooring material, type of vehicle(s) owned, common mode of transportation to school. | same as above | same as above |
| Children’s handwashing | Binary variables: washed hands after using toilet/urinal; used correct technique; used soap; washed hands for correct duration of time | sex | 268 | 363 |
|  | Continuous variables: HW overall score | sex | same as above | same as above |
| Inspection of school WaSH facilities | Binary variables: is clean, is functional, is accessible, is well-let, is not too dark, provides privacy, has no flies, has no signs of graffiti, has no signs of mould, has no signs of damage, has no bad smell, has a garbage can, has a HW basin near toilet/urinal | age of school, primary or secondary grade levels, MOOE budget, location (city) | 86 | 87 |
| Interview with school principals | Binary variables: has policy to clean restrooms daily, has policy to designate a staff member to ensure cleaning policy is enforced; is satisfied with children’s hygiene; is satisfied with school’s sanitation | n/a | 11 | 10 |

BAZ = body mass index-for-age Z score. HAZ = height-for-age Z score. HL = health literacy. HW = handwashing. MOOE = maintenance and other operating expenses. SES = socioeconomic status. SHS = second-hand smoke. WaSH = water, sanitation, and hygiene.

^1^home WaSH = has restroom, shares restroom with another family, restroom is outside of house, has toilet, has HW basin, has faucet with running water.

^2^SES = has electricity, refrigerator, cell phone, computer, clock/watch, electric fan.

^3^food security = enough food, variety of food, can afford to buy food, has asked/begged for food, eats freshly cooked food more often than pre-cooked food.

^4^school WaSH = school restroom is clean; school HW area is clean.

**Additional file 7: Table S3** Surveys conducted during this trial: outcomes, covariates, and sample sizes.

| Outcome | Outcome type | Model | Adjusted for (covariates/ mediators) | Random effect (intercept) | Standard errors | Stata script |
| --- | --- | --- | --- | --- | --- | --- |
| overall HL score, overall germs knowledge score, overall HW knowledge score | continuous | multilevel mixed-effects linear regression | child’s sex & age, parent’s education, SES, outcome at baseline | city | robust | (long file)  preserve  drop if yesigb + yesigc + yesigd + yesigbandd!=0  mixed scorehw_ post##yesiga i.isfemale c.agey_0 c.parenteducode c.ses2 ///  c.scorehwpre\|\| city_0:, vce(robust)  restore |
| overall HL pass, overall germs knowledge pass, overall HW knowledge pass; other HL questions | binary | multilevel mixed-effects logistic regression; multilevel  mixed-effects Poisson regression | child’s sex & age, parent’s education, SES, outcome at baseline | city | robust | (long file)  preserve  drop if yesigb + yesigc + yesigd + yesigbandd!=0  melogit scorehwpass_ post##yesiga i.isfemale c.agey_0 c.parenteducode c.ses2 ///  scorehwpasspre \|\| city_0:, vce(robust) or  restore  or  (long file)  preserve  drop if yesigb + yesigc + yesigd + yesigbandd!=0  mepoisson scorehwpass_ post##yesiga i.isfemale c.agey_0 c.parenteducode c.ses2 ///  scorehwpasspre \|\| city_0:, vce(robust) irr  restore |
| prevalence of: stunting, undernutrition, overnutrition | binary | multilevel mixed-effects logistic regression | child’s sex & age, parent’s education, SES | city | robust | (long file)  preserve  drop if yesigb + yesigc + yesigd + yesigbandd!=0  melogit stunted_ post##yesiga i.isfemale c.agey_0 c.parenteducode c.ses2 ///  \|\| city_0:, vce(robust) or  restore |
| HAZ, BAZ, BMI, height, weight | continuous | multilevel mixed-effects linear regression | child’s sex & age, parent’s education, SES | city | robust | (long file)  preserve  drop if yesigb + yesigc + yesigbandd!=0  mixed haz_ post##yesiga i.isfemale c.agey_0 c.parenteducode c.ses2 ///  \|\| city_0:, vce(robust) |
| Change in HAZ, BAZ, BMI; height & weight gain | continuous | multilevel mixed-effects linear regression | child’s sex & age, parent’s education, SES | city | robust | (widefile)  preserve  drop if yesigb + yesigc + yesigd + yesigbandd!=0  mixed dif_haz i.yesiga i.isfemale c.agey_0 c.parenteducode c.ses2 ///  \|\| city_0:, vce(robust)  restore  and  (widefile)  preserve  drop if yesigb + yesigc + yesigd + yesigbandd!=0  mixed heightgain i.yesiga i.isfemale c.agey_0 c.parenteducode c.ses2 ///  \|\| city_0:, vce(robust)  restore |
| dehydration: mild, moderate, severe, any | binary | multilevel mixed-effects logistic regression; multilevel  mixed-effects Poisson regression | child’s sex & age, SES | city | robust | (long file)  preserve  drop if yesigb + yesigc + yesigd + yesigbandd!=0  melogit yesproteinuria_ post##yesiga i.isfemale c.agey_0 c.ses2 ///  \|\| city_0:, or vce(robust)  restore  or  preserve  drop if yesigb + yesigc + yesigd + yesigbandd!=0  mepoisson veryhiconcurn1030_ post##yesiga i.isfemale c.agey_0 c.ses2 ///  \|\| city_0:, irr vce(robust)  restore |
| urine specific gravity | continuous | multilevel mixed-effects linear regression | child’s sex & age, SES | city | robust | (long file)  preserve  drop if yesigb + yesigc + yesigd + yesigbandd!=0  mixed specificgravitysg_ post##yesiga i.isfemale c.agey_0 c.ses2 ///  \|\| city_0:, vce(robust)  restore |
| observed HW practice: washed hands after using toilet/urinal, used soap, washed for correct duration of time, used correct technique | binary | multilevel mixed-effects logistic regression | female,  primary school, MOOE budget, school restroom has water, HW basin-to-student ratio | city | robust | (wide file)  preserve  drop if yesigb + yesigc + yesigd + yesigbandd!=0  melogit washedhands_1 i.yesiga i.isfemale c.mooe_0 c.studsinkratiov2_0 ///  i.yesprimary c.yeswaternrsc_0 \|\| city:,or vce(robust)  restore |
| observed HW practice score | continuous | multilevel mixed-effects linear regression | female,  primary school, MOOE budget, school restroom has water, HW basin-to-student ratio | city | robust | (wide file)  preserve  drop if yesigb + yesigc + yesigd + yesigbandd!=0  mixed score2modf_1 i.yesiga i.isfemale c.mooe_0 c.studsinkratiov2_0 ///  i.yesprimary c.yeswaternrsc_0 \|\| city:, vce(robust)  restore |
| WaSH adequacy score (of school restrooms) | continuous | multilevel mixed-effects linear regression | school’s annual enrollment,  age of school,  MOOE budget^1^ | city | robust | (long file)  preserve  drop if igb + igc + igd + igbandd!=0  mixed yesfliesnrsc_ post##iga c.enrollallpreo c.ageschoolpreo c.mooepreo ///  \|\| cityid:, vce(robust)  restore |
| Satisfaction of school principals with: school’s sanitation and children’s hygiene | binary | Poisson regression | school’s annual enrollment | n/a | clustered by city | (long file)  preserve  drop if igb + igc + igd + igbandd!=0  poisson yesissatisfiedstudentshygiene_ post##iga c.enrollallpreo, irr vce (cluster cityid)  restore |

BAZ = body mass index-for-age Z score. BMI = body mass index. HAZ = height-for-age Z score. HL = health literacy. HW = handwashing. MOOE = maintenance and other operating expenses. SES = socioeconomic status.

^1^Measurement of covariates during baseline.

**Additional file 8: Table S4** Regression models used to measure intervention effects.

|  | Control | Low-intensity health education | Medium-intensity health education | High-intensity health education | Total |
| --- | --- | --- | --- | --- | --- |
| Characteristics | n (%) | n (%) | n (%) | n (%) | n (%) |
| *Individual level (N = 756)* |  |  |  |  |  |
| Male | 30 (38.5) | 45 (38.8) | 158 (44.4) | 81 (39.3) | 314 (41.5) |
| Adolescent (age ≥ 13 years) | 3 (3.9) | 1 (0.9) | 95 (26.7) | 11 (5.3) | 110 (14.6) |
| *Household factors (N= 828)* | n (%) | n (%) | n (%) | n (%) | n (%) |
| Demographic and caregiver's characteristics |  |  |  |  |  |
| Number of adults in home |  |  |  |  |  |
| 0 | 1 (1.2) | 4 (3.5) | 6 (1.5) | 5 (2.2) | 16 (1.9) |
| 1 to 2 | 27 (31.8) | 45 (39.5) | 128 (32.9) | 74 (31.9) | 279 (33.7) |
| 3 to 4 | 37 (43.5) | 28 (24.6) | 140 (36) | 70 (30.2) | 277 (33.5) |
| 5 or more | 17 (20) | 29 (25.4) | 91 (23.4) | 50 (21.6) | 187 (22.6) |
| > 11 | 3 (3.5) | 8 (7) | 24 (6.2) | 33 (14.2) | 69 (8.3) |
| Number of children in home |  |  |  |  |  |
| 1 to 2 | 37 (43.5) | 65 (57) | 198 (50.9) | 106 (45.7) | 412 (49.8) |
| 3 to 4 | 41 (48.2) | 30 (26.3) | 128 (32.9) | 75 (32.3) | 275 (33.2) |
| 5 to 11 | 6 (7.1) | 14 (12.3) | 53 (13.6) | 37 (15.9) | 110 (13.3) |
| > 11 | 1 (1.2) | 5 (4.4) | 10 (2.6) | 14 (6) | 31 (3.7) |
| Highest level of education completed by parent/caregiver |  |  |  |  |  |
| None | 0 | 0 | 0 | 1 (0.4) | 1 (0.1) |
| Elementary | 2 (2.4) | 6 (5.3) | 22 (5.7) | 23 (9.9) | 53 (6.4) |
| Secondary | 15 (17.6) | 42 (36.8) | 158 (40.6) | 86 (37.1) | 304 (36.7) |
| Vocational | 0 | 1 (0.9) | 16 (4.1) | 11 (4.7) | 28 (3.4) |
| College | 68 (80) | 65 (57) | 193 (49.6) | 111 (47.8) | 442 (53.4) |
| Number of adults who are employed, median (IQR) | 2 (2 - 3) | 2 (1 - 3) | 2 (1 - 3) | 2 (1 - 3) | 2 (0 - 8) |
| 0 | 2 (2.4) | 5 (4.4) | 12 (3.1) | 12 (5.2) | 31 (3.7) |
| 1 | 17 (20) | 33 (28.9) | 117 (30.1) | 48 (20.7) | 219 (26.4) |
| 2 | 35 (41.2) | 41 (36) | 128 (32.9) | 84 (36.2) | 290 (35) |
| 3 to 4 | 25 (29.4) | 24 (21.1) | 94 (24.2) | 56 (24.1) | 200 (24.2) |
| 5 or more | 6 (7.1) | 11 (9.6) | 38 (9.8) | 32 (13.8) | 88 (10.6) |
| Number of children who are employed, median (IQR) | 0 (0 - 0) | 0 (0 - 0) | 0 (0 - 0) | 0 (0 - 0.5) | 0 (0 - 5) |
| 0 | 75 (88.2) | 99 (86.8) | 335 (86.1 ) | 174 (75) | 691 (83.5) |
| 1 | 6 (7.1) | 4 (3.5) | 26 (6.7) | 21 (9.1) | 57 (6.9) |
| 2 | 2 (2.4) | 3 (2.6) | 15 (3.9) | 10 (4.3) | 30 (3.6) |
| 3 to 4 | 2 (2.4) | 6 (5.3) | 8 (2.1 ) | 15 (6.5) | 31 (3.7) |
| 5 or more | 0 | 2 (1.8) | 5 (1.3 ) | 12 (5.2) | 19 (2.3) |
| Someone at home is a smoker | 31 (36.5) | 52 (45.6) | 190 (48.8 ) | 117 (50.4) | 394 (47.6) |
| Someone smokes inside home | 9 (10.6) | 26 (22.8) | 94 (24.2) | 74 (31.9) | 204 (24.6) |
| Parent/caregiver is person with disability | 4 (4.7) | 8 (7) | 30 (7.7 ) | 18 (7.8) | 61 (7.4) |
| Parent/caregiver cannot read | 0 | 1 (0.9) | 12 (3.1) | 8 (3.4) | 21 (2.5) |
| *Socioeconomic characteristics (N = 828)* |  |  |  |  |  |
| Material of home’s flooring |  |  |  |  |  |
| Cement | 36 (42.4) | 57 (50) | 199 (51.2) | 116 (50) | 412 (49.8) |
| Ceramic | 40 (47.1) | 54 (47.4) | 136 (35 ) | 87 (37.5) | 320 (38.6) |
| Sand/soil | 1 (1.2) | 1 (0.9) | 3 (0.8) | 4 (1.7) | 10 (1.2) |
| Wood | 8 (9.4) | 2 (1.8) | 51 (13.1) | 25 (10.8) | 86 (10.4) |
| No electricity | 0 | 0 | 3 (0.8) | 1 (0.4) | 4 (0.5) |
| No cell phone | 2 (2.4) | 7 (6.1) | 29 (7.5) | 21 (9.1) | 60 (7.2) |
| No computer | 32 (37.6) | 71 (62.3) | 249 (64) | 140 (60.3) | 496 (59.9) |
| No watch/clock | 3 (3.5) | 5 (4.4) | 25 (6.4) | 13 (5.6) | 47 (5.7) |
| No electric fan | 0 | 1 (0.9) | 4 (1) | 4 (1.7) | 9 (1.1) |
| No vehicle | 26 (30.6) | 38 (33.3) | 161 (41.4) | 99 (42.7) | 328 (39.6) |
| Bicycle/pedicab only | 4 (4.7) | 7 (6.1) | 50 (12.9) | 34 (14.7) | 97 (11.7) |
| Tricycle/scooter/motorcycle only | 14 (16.5) | 43 (37.7) | 109 (28) | 57 (24.6) | 225 (27.2) |
| Car only | 38 (44.7) | 18 (15.8) | 53 (13.6) | 28 (12.1) | 137 (16.5) |
| *Food insecurity factors (N =828)* |  |  |  |  |  |
| Not enough food | 0 | 1 (0.9) | 13 (3.3) | 13 (5.6) | 27 (3.3) |
| Does not eat variety of food | 1 (1.2) | 4 (3.5) | 19 (4.9) | 10 (4.3) | 34 (4.1) |
| Often cannot afford to buy food | 0 | 4 (3.5) | 17 (4.4) | 8 (3.4) | 29 (3.5) |
| Often has asked/begged for food | 26 (30.6) | 33 (28.9) | 82 (21.1) | 56 (24.1) | 198 (23.9) |
| Eats pre-cooked food more often than freshly cooked food | 11 (12.9) | 13 (11.4) | 36 (9.3) | 20 (8.6) | 82 (9.9) |
| *WaSH-related factors (N = 828)* |  |  |  |  |  |
| Source of drinking water in home |  |  |  |  |  |
| Faucet only | 9 (10.6) | 35 (30.7) | 70 (18) | 49 (21.1) | 163 (19.7) |
| Filtered only | 16 (18.8) | 25 (21.9) | 104 (26.7) | 59 (25.4) | 206 (24.9) |
| Bottled/from water refilling station only | 48 (56.5) | 38 (33.3) | 189 (48.6) | 104 (44.8) | 385 (46.5) |
| Any bottled water | 57 (67.1) | 53 (46.5) | 214 (55) | 121 (52.2) | 451 (54.5) |

IQR = interquartile range. WaSH = water, sanitation, and hygiene.

Percentages were calculated from smaller denominators than those shown at the top of the table for all variables because of missing values.

**Additional file 9: Table S5** Expanded table: characteristics of study population by study arm.

| **Outcome** | **Study arm** | **Baseline** | | **Endline** | | **Effect of intervention  (95% CI)** | **p-value** |
| --- | --- | --- | --- | --- | --- | --- | --- |
|  |  | n | % | n | % |  |  |
| **Knows when to wash hands*** | Control | 62 | 79.5 | 78 | 96.3 |  |  |
|  | Low-intensity education | 99 | 97.6 | 96 | 97 | 0.92 (0.70, 1.22) | 0.57 |
|  | Medium-intensity education | 296 | 85.6 | 290 | 96.4 | 0.99 (0.80, 1.22) | 0.90 |
|  | High-intensity education | 159 | 77.6 | 169 | 93.9 | 1.05 (0.82, 1.36) | 0.68 |
| **Knows how long to wash hands*** | Control | 26 | 33.3 | 46 | 56.8 |  |  |
|  | Low-intensity education | 46 | 40.7 | 83 | 83.8 | 1.13 (0.63, 2.04) | 0.68 |
|  | Medium-intensity education | 86 | 24.9 | 135 | 44.9 | 1.17 (0.71, 1.92) | 0.53 |
|  | High-intensity education | 73 | 35.6 | 111 | 61.7 | 1.06 (0.19, 5.84) | 0.95 |
| **Knows how to prevent infection by washing hands*** | Control | 75 | 96.2 | 77 | 95.1 |  |  |
|  | Low-intensity education | 109 | 96.5 | 97 | 98 | 1.06 (1.00, 1.12) | 0.07 |
|  | Medium-intensity education | 316 | 91.3 | 285 | 94.7 | 1.10 (1.04, 1.15) | p < 0.01 |
|  | High-intensity education | 200 | 97.6 | 174 | 96.7 | 1.03 (0.97, 1.09) | 0.38 |
| **Knows that germs are disease-causing organisms*** | Control | 76 | 97.4 | 80 | 98.8 |  |  |
|  | Low-intensity education | 109 | 96.5 | 99 | 100 | 0.98 (0.95, 1.01) | 0.15 |
|  | Medium-intensity education | 334 | 96.5 | 296 | 98.3 | 0.99 (0.95, 1.03) | 0.66 |
|  | High-intensity education | 192 | 93.7 | 174 | 96.7 | 1.02 (0.96, 1.09) | 0.49 |
| **Knows where germs may be found*** | Control | 58 | 74.4 | 78 | 96.3 |  |  |
|  | Low-intensity education | 90 | 79.7 | 97 | 98 | 0.83 (0.49, 1.42) | 0.50 |
|  | Medium-intensity education | 282 | 81.5 | 288 | 95.7 | 0.83 (0.54, 1.27) | 0.39 |
|  | High-intensity education | 154 | 75.1 | 165 | 91.7 | 0.87 (0.58, 1.30) | 0.49 |
| **Knows that germs can be removed with soap* and water** | Control | 71 | 91 | 80 | 98.8 |  |  |
|  | Low-intensity education | 104 | 92 | 97 | 98 | 1.01 (0.98, 1.04) | 0.48 |
|  | Medium-intensity education | 311 | 89.9 | 293 | 97.3 | 1.06 (0.99, 1.13) | 0.09 |
|  | High-intensity education | 176 | 85.9 | 174 | 96.7 | 1.13 (1.07, 1.20) | p < 0.01 |
| **Knows that a restroom that is not clean may contain germs*** | Control | 59 | 75.6 | 62 | 76.5 |  |  |
|  | Low-intensity education | 111 | 98.2 | 91 | 91.9 | 0.97 (0.63, 1.50) | 0.90 |
|  | Medium-intensity education | 211 | 61 | 208 | 69.1 | 1.21 (0.81, 1.80) | 0.36 |
|  | High-intensity education | 170 | 82.9 | 156 | 86.7 | 1.09 (0.75, 1.57) | 0.66 |
| **Knows to wash fruits and vegetables before eating to remove germs and prevent infection*** | Control | 67 | 85.9 | 70 | 86.4 |  |  |
|  | Low-intensity education | 107 | 94.7 | 92 | 92.9 | 1 (0.81, 1.24) | 0.98 |
|  | Medium-intensity education | 302 | 87.3 | 261 | 86.7 | 1.04 (0.88, 1.23) | 0.67 |
|  | High-intensity education | 197 | 96.1 | 165 | 91.7 | 0.97 (0.81, 1.16) | 0.73 |
| **Knows how germs can be spread from person to person*** | Control | 62 | 79.5 | 77 | 95.1 |  |  |
|  | Low-intensity education | 88 | 77.9 | 98 | 99 | 1.06 (0.84, 1.33) | 0.65 |
|  | Medium-intensity education | 293 | 84.7 | 284 | 94.4 | 1.01 (0.85, 1.20) | 0.92 |
|  | High-intensity education | 161 | 78.5 | 166 | 92.2 | 1.07 (0.89, 1.28) | 0.48 |
| **Knows primary symptoms of infection*** | Control | 74 | 94.9 | 77 | 95.1 |  |  |
|  | Low-intensity education | 109 | 96.5 | 97 | 98 | 1.02 (0.93, 1.12) | 0.62 |
|  | Medium-intensity education | 315 | 91 | 278 | 92.4 | 1.04 (0.96, 1.11) | 0.35 |
|  | High-intensity education | 194 | 94.6 | 173 | 96.1 | 1.01 (0.94, 1.09) | 0.72 |
| **Knows secondary symptoms of infection*** | Control | 37 | 72.6 | 27 | 84.4 |  |  |
|  | Low-intensity education | 105 | 92.9 | 62 | 98.4 | 0.82 (0.81, 0.83) | p < 0.01 |
|  | Medium-intensity education | 120 | 80 | 42 | 97.7 | 1.15 (1.07, 1.24) | p < 0.01 |
|  | High-intensity education | 123 | 81.5 | 67 | 87 | 0.82 (0.63, 1.07) | 0.14 |
| **Washed hands with soap**** | Control |  |  | 2 | 11.1 |  |  |
|  | Low-intensity education |  |  |  |  |  |  |
|  | Medium-intensity education |  |  | 1 | 6.7 | 1.75 (1.75, 1.75) | p < 0.01 |
|  | High-intensity education |  |  | 2 | 5 | 0.46 (0.13, 1.66) | 0.24 |
| **Used correct handwashing technique**** | Control |  |  | 8 | 44.4 |  |  |
|  | Low-intensity education |  |  | 1 | 6.7 | 0.10 (0.07, 0.15) | p < 0.01 |
|  | Medium-intensity education |  |  | 3 | 20 | 11 (5.53, 21.7) | p < 0.01 |
|  | High-intensity education |  |  | 3 | 7.5 | 0.12 (0.10, 0.15) | p < 0.01 |

aOR = adjusted odds ratio. CG = control group. CI = confidence interval. IG = intervention group. IRR = incidence-rate ratio. MOOE = maintenance and other operating expenses. SES = socioeconomic status.

The p-value refers to the difference in intervention effect between the respective IG and the CG.

*We used a multilevel mixed-effects Poisson regression model to estimate intervention effects, which can be interpreted as the IRR of a desired follow-up outcome between the respective IG and the CG. The model included the respective IG, random intercept for the city, and robust standard errors. We adjusted for the child’s sex, age, and desired outcome at baseline, and the parent/caregiver’s education level and SES.

**We used a multilevel mixed-effects logistic regression model to estimate intervention effects expressed as the aOR of the prevalence at endline of the desired outcome between the respective IG and CG. The model included the respective IG, random intercept for the city, and robust standard errors. We adjusted for the child’s sex, attendance in primary school, the school’s MOOE budget and handwashing basin-to-student ratio, and the availability of water in the school restroom.

**Additional file 10: Table S6** Expanded table: effect of intervention on children’s hygiene-related health literacy and observed handwashing.

| **Outcome** | | **Study arm** | **Baseline** | | **Endline** | | **Effect of intervention (95% CI)** | **p-value** |
| --- | --- | --- | --- | --- | --- | --- | --- | --- |
|  |  |  | **n** | **%** | **n** | **%** |  |  |
| **Undernutrition** | **Severe thinness** | Control | 4 | 5.1 | 3 | 3.7 |  |  |
|  |  | Low-intensity education | 5 | 4.8 | 3 | 2.9 | * |  |
|  |  | Medium-intensity education | 21 | 6 | 18 | 5.8 | * |  |
|  |  | High-intensity education | 7 | 3.4 | 10 | 5.5 | * |  |
|  | **Thinness** | Control | 2 | 2.6 | 0 | 0 |  |  |
|  |  | Low-intensity education | 3 | 2.9 | 3 | 2.9 | * |  |
|  |  | Medium-intensity education | 10 | 2.8 | 6 | 1.9 | * |  |
|  |  | High-intensity education | 6 | 2.9 | 3 | 1.7 | * |  |
| **Overnutrition** | **Overweight** | Control | 25 | 32.1 | 19 | 23.5 |  |  |
|  |  | Low-intensity education | 27 | 26 | 18 | 17.3 | 0.80 (0.47, 1.36) | 0.41 |
|  |  | Medium-intensity education | 73 | 20.7 | 44 | 14.2 | 0.72 (0.39, 1.31) | 0.28 |
|  |  | High-intensity education | 44 | 21.4 | 27 | 14.9 | 0.64 (0.42, 0.98) | 0.04 |
|  | **Obesity** | Control | 2 | 2.6 | 17 | 21 |  |  |
|  |  | Low-intensity education | 5 | 4.8 | 13 | 12.5 | 0.52 (0.37, 0.72) | p < 0.01 |
|  |  | Medium-intensity education | 3 | 0.9 | 24 | 7.7 | 0.78 (0.49, 1.23) | 0.28 |
|  |  | High-intensity education | 0 | 0 | 11 | 6.1 | 0.76 (0.59, 0.97) | 0.03 |

aOR = adjusted odds ratio. BAZ = body mass index-for-age Z score. BMI = body mass index. CG = control group. CI = confidence interval. cm = centimetre. HAZ = height-for-age Z score. IG = intervention group. kg = kilogram.

The p-value refers to the difference in intervention effect between the respective IG and the CG. We classified nutrition status using the 2007 WHO Growth Reference. Stunting = HAZ < −2 SD. Undernutrition = composite variable comprised of thinness (−3 < BAZ < −2) and severe thinness (BAZ < −3). Overnutrition = composite variable comprised of overweight (1 < BAZ < 2) and obesity (BAZ > 2).

*No result.

**We used a multilevel mixed-effects logistic regression model to estimate intervention effects, which can be expressed as the aOR of change in prevalence of a desired follow-up outcome between the respective IG and the CG. The model included the respective IG, random intercept for the city, and robust standard errors. We adjusted for the child’s sex and age, and the parent/caregiver’s education level and socioeconomic status SES.

**Additional file 11: Table S7** Expanded table: effect of intervention on children’s malnutrition status.

| **Outcome** | **Study arm** | **Endline mean (± SD)** | **Effect of intervention (95% CI)** | **p-value** |
| --- | --- | --- | --- | --- |
| **Change in HAZ (stunting)** | Control | -0.26 (0.67) |  |  |
|  | Low-intensity education | -0.02 (1.09) | 0.23 (-0.04, 0.49) | 0.09 |
|  | Medium-intensity education | 0.05 (0.54) | 0.21 (-0.13, 0.55) | 0.22 |
|  | High-intensity education | -0.03 (0.45) | 0.15 (-0.04, 0.34) | 0.12 |
| **Change in BAZ (thinness)** | Control | 0.12 (0.68) |  |  |
|  | Low-intensity education | -0.02 (0.94) | -0.22 (-0.35, -0.09) | p < 0.01 |
|  | Medium-intensity education | 0.09 (0.73) | -0.04 (-0.25, 0.17) | 0.72 |
|  | High-intensity education | 0.06 (0.59) | -0.05 (-0.28, 0.18) | 0.68 |
| **Change in BMI** | Control | 0.86 (1.78) |  |  |
|  | Low-intensity education | 0.14 (4.58) | -0.99 (-1.67, -0.31) | p < 0.01 |
|  | Medium-intensity education | 0.62 (1.37) | -0.22 (-0.79, 0.35) | 0.45 |
|  | High-intensity education | 0.58 (1.25) | -0.21 (-0.62, 0.21) | 0.33 |
| **Height (cm) gain** | Control | 1.52 (5.08) |  |  |
|  | Low-intensity education | 3.65 (1.67) | 2.46 (0.39, 4.53) | 0.02 |
|  | Medium-intensity education | 3.97 (3.92) | 3.62 (1.43, 5.81) | p < 0.01 |
|  | High-intensity education | 3.17 (3.28) | 3.02 (1.80, 4.25) | p < 0.01 |
| **Weight (kg) gain** | Control | 2.84 (2.85) |  |  |
|  | Low-intensity education | 2.90 (2.76) | 0.14 (-0.39, 0.67) | 0.60 |
|  | Medium-intensity education | 3.37 (3.22) | 0.52 (0.44, 0.59) | p < 0.01 |
|  | High-intensity education | 2.85 (2.73) | 0.23 (-0.49, 0.94) | 0.54 |

BAZ = body mass index-for-age Z score. BMI = body mass index. CG = control group. CI = confidence interval. cm = centimetre. HAZ = height-for-age Z score. IG = intervention group. kg = kilogram. SD = standard deviation. SES = socioeconomic status.

The p-value refers to the difference in intervention effect between the respective IG and the CG.

*We used a multilevel mixed-effects linear regression model to estimate intervention effects, which can be interpreted as the adjusted differences in the mean changes of the desired follow-up outcome between the respective IG and the CG. The model included the respective IG, random intercept for the city, and robust standard errors. We adjusted for the child’s sex and age and the parent/caregiver’s education level and SES.

**Additional file 12: Table S8** Effect of the intervention on changes in children’s growth indicators.

| **Outcome** | **Study arm** | **Baseline** | | **Endline** | | **Effect of intervention  (95% CI)** | **p-value** |
| --- | --- | --- | --- | --- | --- | --- | --- |
|  |  | n | % | n | % |  |  |
| **Any* dehydration** | Control | 69 | 89.6 | 76 | 97.4 |  |  |
|  | Low-intensity education | 91 | 88.4 | 97 | 93.3 | 0.46 (0.04, 5.21)** | 0.53 |
|  | Medium-intensity education | 305 | 90.2 | 272 | 82.9 | 0.14 (0.01, 1.72)** | 0.12 |
|  | High-intensity education | 184 | 89.8 | 160 | 88.9 | 0.24 (0.01, 6.34)** | 0.39 |
| **Urine specific gravity** | Control | 1.027 | 0.006 | 1.028 | 0.004 |  |  |
|  | Low-intensity education | 1.026 | 0.006 | 1.028 | 0.006 | -0.000 (-0.001, 0.000)*** | 0.13 |
|  | Medium-intensity education | 1.026 | 0.006 | 1.025 | 0.008 | -0.004 (-0.004, -0.004)*** | p < 0.01 |
|  | High-intensity education | 1.026 | 0.006 | 1.026 | 0.007 | -0.002 (-0.005, 0.002)*** | 0.33 |

aOR = adjusted odds ratio. CG = control group. CI = confidence interval. IG = intervention group. SES = socioeconomic status. U_sg_ = urine specific gravity.

The p-value refers to the difference in intervention effect between the respective IG and the CG.

*We defined any dehydration as U_sg_ ≥ 1.020.

**We used a multilevel mixed-effects logistic regression model to estimate intervention effects, which can be expressed as the aOR of change in prevalence of a desired follow-up outcome between the respective IG and CG. The model included the respective IG, random intercept for the city, and robust standard errors. We adjusted for the child’s sex, age, and SES.

***We used a multilevel mixed-effects linear regression model to estimate intervention effects, which can be interpreted as the adjusted differences in the mean changes of the desired outcome between the respective IG and the CG. The model included the respective IG, random intercept for the city, and robust standard errors. We adjusted for the child’s sex, age, and SES.

**Additional file 13: Table S9** Expanded table: effect of intervention on children’s dehydration status and urine specific gravity.

Page left intentionally blank.
